# Easy Ligand Activation in the Coordination Sphere of Ru inside the [PW_11_O_39_]^7–^ Backbone

**DOI:** 10.3390/molecules25081859

**Published:** 2020-04-17

**Authors:** Anna A. Mukhacheva, Artem L. Gushchin, Vadim V. Yanshole, Pavel A. Abramov, Maksim N. Sokolov

**Affiliations:** 1 Nikolaev Institute of Inorganic Chemistry, Siberian Branch of Russian Academy of Sciences, 3 Akad. Lavrentiev Ave., 630090 Novosibirsk, Russia; anna-mukhacheva@mail.ru (A.A.M.); gushchin_al@yahoo.com (A.L.G.); abramov@niic.nsc.ru (P.A.A.); 2 International Tomography Center SB RAS, Institutskaya str. 3a, 630090 Novosibirsk, Russia; vadim.yanshole@tomo.nsc.ru; 3 Novosibirsk State University, Pirogova str. 2, 630090 Novosibirsk, Russia; 4 South Ural State University, Prospekt Lenina 76, 454080 Chelyabinsk, Russia

**Keywords:** ruthenium, polyoxometalate, azide, CV, HR-ESI-MS.

## Abstract

Irradiation of the Keggin-type [PW_11_O_39_{Ru(NO)}]^4−^ (**Ru-NO**) polyoxometalate in CH_3_CN results in rapid NO ligand elimination with the formation of [PW_11_O_39_{Ru^III^(CH_3_CN)}]^4−^ (**Ru-CH_3_CN**). This complex offers an easy entry into the Ru-based chemistry of the {PW_11_Ru} complex. Attempts to substitute N_3_^−^ for CH_3_CN in the presence of an NaN_3_ excess lead a variety of products: (i) [PW_11_O_39_{Ru^III^(N_3_)}]^4−^ (**Ru-N_3_**); (ii) [PW_11_O_39_{Ru^III^(N_4_HC-CH_3_)}]^4−^ (**Ru-Tz**) as a click-reaction product; and (iii) [PW_11_O_39_{Ru^II^(N_2_)}]^5−^ (**Ru-N_2_**). UV-VIS, CV, and HR-ESI-MS techniques were used for the reaction monitoring and characterization of the products.

## 1. Introduction

Chemistry of noble-metal-substituted polyoxometalates, apart from general chemical interest of bringing noble metal centers in coordination to what can be regarded as noninnocent multidentate oxygen-donor ligand, is important for the search of new pathways of coordinated molecules activation and design of new catalysts, including photo- and electrocatalysts.

Of the polyoxometalates (POMs) incorporating noble metals, the most studied are Ru-substituted Keggin-type heteropolytungstates, where a {W=O}^4+^ moiety in the plenary Keggin archetype, [XW_12_O_40_]^n−^ (X = P (*n* = 3), Si and Ge (*n* = 4)), is substituted by a {RuL} group. L can be neutral or charged, such as O^2−^ or N^3−^, and Ru in POMs is known to exist in at least five oxidation states, ranging from Ru(II) to Ru(VI). Incorporation of Ru confers unique redox and catalytic properties on the resulting POMs [1,2]. Catalytic activities of [PW_11_O_39_Ru^III^(H_2_O)]^4−^, [PW_11_O_39_Ru^II^(DMSO)]^5−^ (DMSO: dimethyl sulfoxide), [SiW_11_O_39_Ru^III^(H_2_O)]^5−^, [SiW_11_O_39_Ru^III^(DMSO)]^5−^, and [GeW_11_O_39_Ru^III^(H_2_O)]^5−^ in oxidation of olefins [3,4,5], water [6,7,8], DMSO [9,10], and alcohols [11,12]; reduction of DMSO [10] and carbon dioxide [13]; and oxidative C–C bond formation [14] have been reported. The H_2_O ligand attached to Ru is exchangeable with other organic and inorganic donor molecules to form **Ru-pyridine** [10,15,16], **Ru-pyrazine** [12], Ru-DMSO [10,17,18], **Ru-NO** [19,20,21], **Ru-Cl** [22], **Ru-CO** [23,24,25], **Ru-olefin** [10], and **Ru-O-Ru** derivatives [26,27].

In 2013 we reported incorporation of a {Ru(NO)}^3+^ group into a Keggin type POM, [PW_11_O_39_]^7−^, and versatile reactivity of the coordinate NO ligand [20,21]. Easily available [Ru(NO)Cl_5_]^2−^ offers serious advantages as Ru source for preparation of Ru-substituted Keggin-type heteropolytungstates due to the redox stability of the {Ru(NO)}^3+^ unit, hydrolytic inertness, and consequent lack of uncontrollable hydrolytic oligomerization, though the inertness of coordinated Cl ligands in this low-spin d^6^-Ru(II) complex requires drastic reaction conditions. The coordinated NO can be destroyed or eliminated, leaving a vacant coordination site, which can open a way for activation of different substrates in catalytical applications [28,29,30]. Recently, we reported the reactions of [Ru(NO)Cl_5_]^2−^ with pseudotrivacant B-α-[XW_9_O_33_]^9−^ (X = As^III^, Sb^III^) at 160 °C resulting in rearrangement of the polyoxometalate backbones into {XM_18_} structures [31]. In the case of [α-B-AsW_9_O_33_]^9−^, oxidation of As(III) to As(V) took place, accompanied by rearrangement into Dawson type [As_2_W_17_{Ru(NO)}O_61_]^7−^. In the case of [α-B-SbW_9_O_33_]^9−^, the product [SbW_17_{Ru(NO)}O_59_]^10−^ was isolated as (DMAH)_10_[SbW_17_{Ru(NO)}O_59_]∙11H_2_O.

Another aspect highlighted here is reactions of coordinated ligands [32] or ligand activation in the coordination sphere of noble metals. Numerous research papers have been published in this field. Specifically we would like to highlight the reactivity of coordinated nitriles in the Pt coordination sphere [33,34,35,36,37] or reactivity of triosmium clusters toward a wide range of substrates [38,39,40,41]. UV-irradiation (or photolysis) is a well-known technique for a lot of chemical processes, including (i) ligand exchange reactions (e.g., low-valence iron, osmium clusters) [42,43], (ii) ligand transformation [44,45,46], and (iii) CO_2_ capture [47].

Here, we report an easy way to remove the NO ligand from [PW_11_O_39_(Ru(NO)}^4−^, quantitative generation of [PW_11_O_39_Ru^III^(CH_3_CN)]^4−^, and its reactions with N_3_^–^ which follow three different pathways.

## 2. Results

Coordinated NO (formally, NO^+^) ligand in the {Ru(NO)}^3+^ complexes typically can play three roles: (i) stabilizing low-spin d^6^-Ru(II) and imparting stability and inertness on the coordination sphere; (ii) acting as spectator ligand, capable of taking up or releasing extra electron density on Ru due to π-backbonding; (iii) easy transformations or elimination (releasing a coordination site) upon irradiation or in the presence of reducing agents or nucleophiles. In this work we used mercury lamp irradiation to eliminate NO ligand from [PW_11_O_39_{Ru(NO)}]^4−^ (**Ru-NO**) (Figures S1–S3, Table S1) in CH_3_CN solution producing corresponding [PW_11_O_39_{Ru^III^(CH_3_CN)}]^4−^ anion. The reaction can be viewed as dissociation of neutral NO molecule with the formation of coordinatively unsaturated species [PW_11_O_39_Ru^III^]^4−^, followed by rapid solvation with CH_3_CN, quantitatively yielding [PW_11_O_39_{Ru^III^(CH_3_CN)}]^4−^ (**Ru-CH_3_CN)**. This process was followed by IR, UV-VIS, and CV techniques.

**Figure 1:**
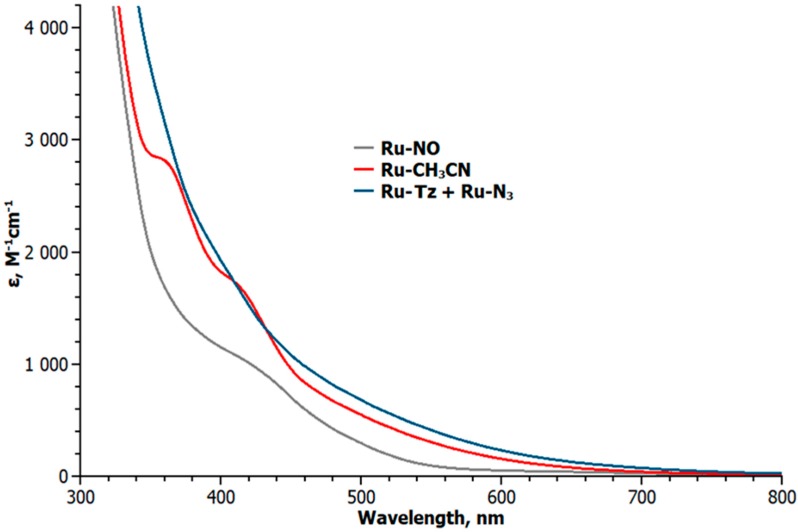
UV-VIS spectra for POM-Ru derivatives.

**Figure 2:**
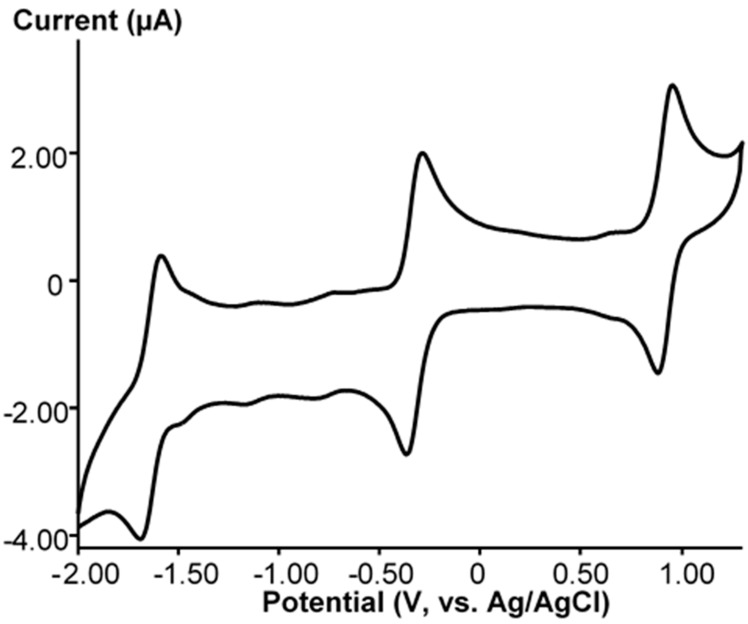
CV of **Ru-CH_3_CN** in CH_3_CN at scan rate of 100 mV/s.

After 90 min irradiation of a solution of **Ru-NO** in CH_3_CN, the UV-VIS spectrum changed (Figure 1) by disappearance of the band at 412 nm (ε = 1059 M^–1^cm^–1^), and appearance of two new bands at 356 nm (ε = 2843 M^−1^cm^−1^) and 410 nm (ε = 1720 M^−1^cm^−1^). Cyclic voltammetry (CV) can be used for the monitoring of the substitution of CH_3_CN for NO by following the change in the corresponding redox processes. The CV plot of **Ru-CH_3_CN** in acetonitrile is shown in Figure 2. Three redox processes at E_1/2_ = 0.92, −0.33 and −1.64 V (vs. Ag/AgCl) were detected. The difference between the anodic and cathodic peak potentials (ΔE) does not exceed 80 mV, and the ratio between the anodic and cathodic peak currents (I_a_/I_c_) is close to 1 for each of these processes. All this indicates the reversibility of the processes. The oxidation process at 0.92 V corresponds to Ru(II)/Ru(III) couple. It was previously found that a similar **Ru-NO** complex reversibly oxidizes at 1.29 V (vs. Ag/AgCl) [20]. Two redox processes at −0.33 and −1.64 V should correspond to the reduction of W(VI) in the {PW_11_} framework, as is well documented.

The **Ru-CH_3_CN** complex can be isolated as TBA-salt by diethyl ether diffusion for crystalline sample or bulk ether precipitation of the crude product. IR-spectroscopy confirms absence of NO ligand by disappearance of the tell-tale NO band at 1846 cm^−1^ (Figures S7 and S8). 

**Figure 3:**
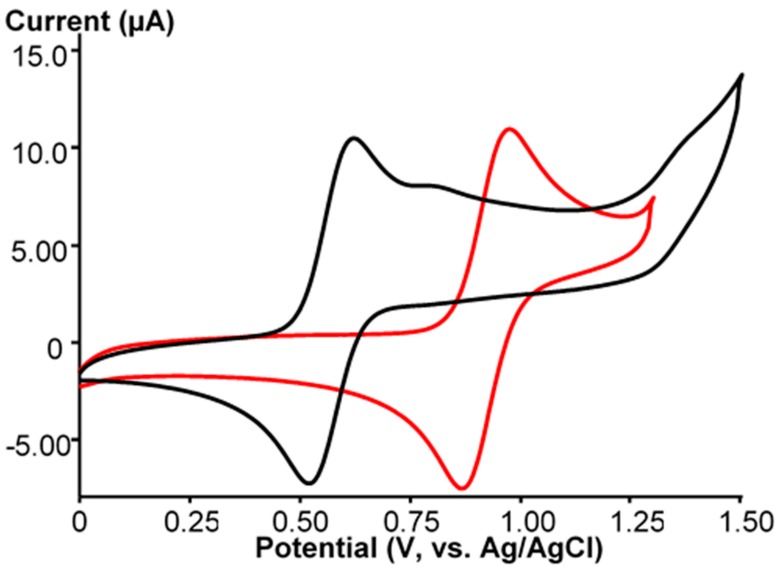
CVs of **Ru-N_3_**/**Ru-Hc** (black line) and **Ru-CH_3_CN** (red line) in CH_3_CN in the range from 0 to 1.50 V at potential scan rate of 100 mV/s.

**Figure 4:**
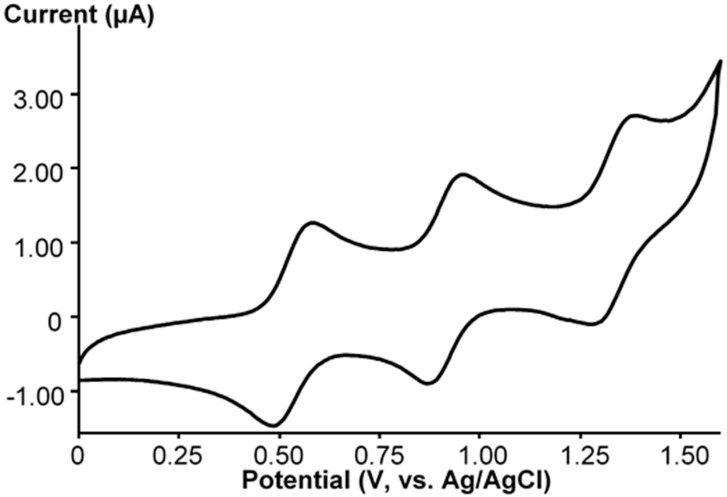
CV of the reaction mixture in CH_3_CN in the range from 0 to 1.60 V at potential scan rate of 100 mV/s.

**Ru-CH_3_CN** was prepared to study reactivity at the Ru site in various reactions. We tried to exchange the coordinated acetonitrile molecule with azide ligand in order to make the [PW_11_O_39_Ru^III^(N_3_)]^5−^ (**Ru-N_3_**) complex and to study the reactions of the coordinated azide ligand. The reaction was monitored by CV. Heating of **Ru-CH_3_CN** with a large excess of NaN_3_ results in the changing of the UV-VIS spectrum (Figure 1). The spectrum differs from that of **Ru-CH_3_CN** or **Ru-NO** and looks essentially featureless. The implicit absorption bands could be identified by calculating wavelength derivatives [48]. Two bands at 366 nm (ε = 2888 M^−1^cm^−1^) and 415 nm (ε = 1633 M^−1^cm^−1^) were detected (Figure S9). Substitution of acetonitrile leads to a noticeable cathodic shift in the Ru(II)/Ru(III) potential from 0.92 V (red line in Figure 3) to 0.57 V (black line in Figure 3). Additionally two small irreversible shoulders were detected. However, within the concentrations of NaN_3_ employed in this work, we were unable to achieve complete substitution of CH_3_CN by azide by working in CH_3_CN. However, with large excess in NaN_3_, the cyclic voltammogram plot shows the presence of three reversible Ru(II)/Ru(III) waves in the positive region (Figure 4). The first one corresponds to the formation of the azide complex, and the second one corresponds to the unreacted acetonitrile complex.

**Figure 5:**
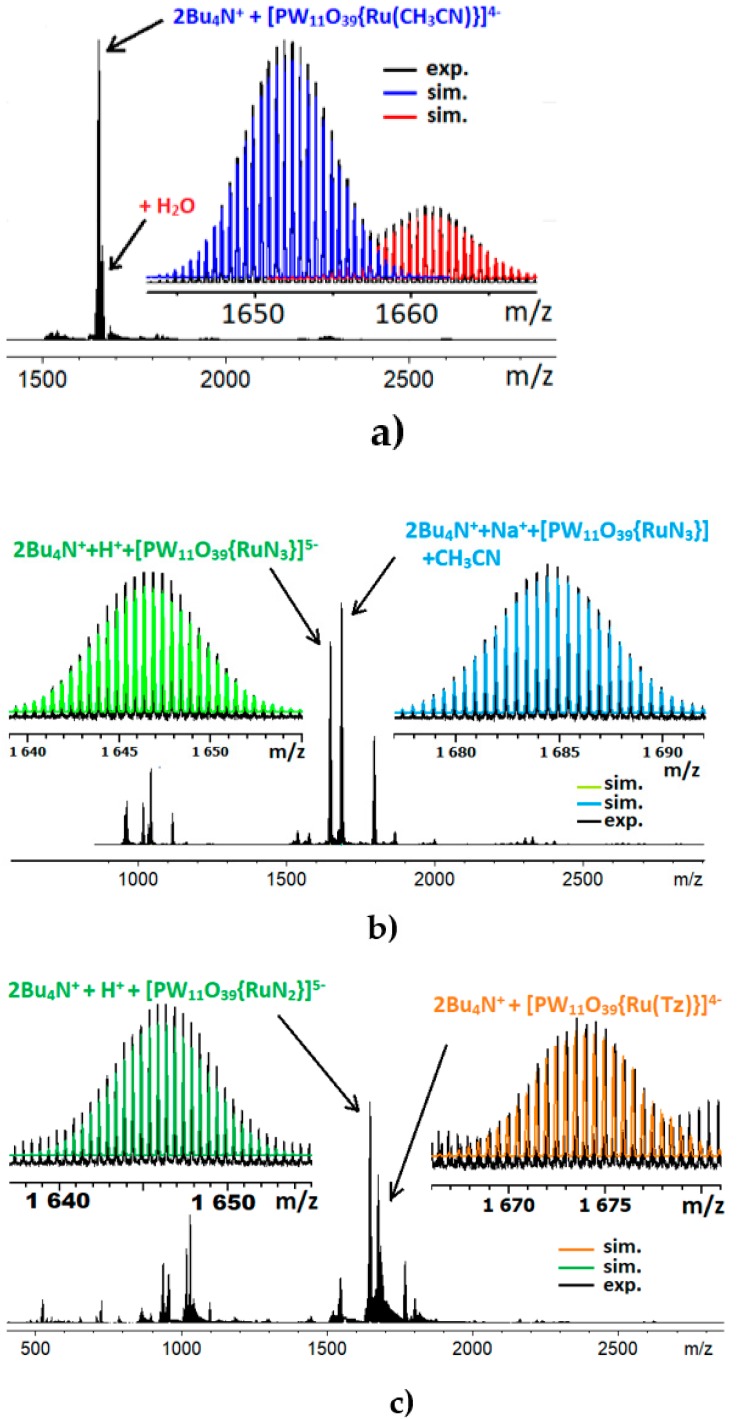
HR-ESI-MS spectra of **Ru-CH_3_CN** (**a**), **Ru-N_3_** (**b**), **Ru-N_2_** and **Ru-Tz** (**c**).

The composition of the complex was confirmed with elemental analysis and HR-ESI-MS (Figure 5, Figures S4–S6, Table S2). We also used HR-ESI-MS techniques to study these reactions. The products were isolated by diethyl ether precipitation as mixtures of TBA salts and redissolved in CH_3_CN for the measurements. The spectra (Figure 5, Figures S10–S15, Table S3) indicate the presence of both **Ru-N_3_** and **Ru-CH_3_CN** complexes. Moreover, there are other species, such as {2H^+^ + **Ru-N_3_** + CH_3_CN}^3−^ (*m*/*z* 954.451), {Na^+^ + H^+^ + **Ru-N_3_** + CH_3_CN}^3−^ (*m*/*z* 961.779), or {2Bu_4_N^+^ + **Ru-N_3_** + CH_3_CN}^3−^ (*m*/*z* 1115.424) (Table S3). Since an acetonitrile molecule is routinely required for matching calculated and experimental *m*/*z* values, this can be attributed to the formation of N_4_HC-CH_3_ (**Tz**) tetrazole or tetrazolate (**Tz^−^**) in the coordination sphere of ruthenium as a click reaction result ([2 + 3] dipolar addition of azide to the nitrile bond). We have to conclude that the formation of **Ru-N_3_** and [PW_11_O_39_{Ru^III^(N_4_HC-CH_3_)}]^4−^ (**Ru-Tz**) anion occurred during the reaction.

When the reaction is carried out in a weakly coordinating CH_3_NO_2_, the HR-ESI-MS data (Figure 5, Figures S16–S21, Table S4) reflect different pathways of ligand transformation. There are two products, with one of them being the above-mentioned **Ru-Tz** complex. This [PW_11_O_39_{Ru^III^(N_4_HC-CH_3_)}]^4−^ (**Ru-Tz**) was detected in the composite peaks {2Bu_4_N^+^ + **Ru-Tz**}^2−^ (*m*/*z* 1673.633) and {2Bu_4_N^+^ + **Ru-Tz** + H_2_O}^2−^ (*m*/*z* 1682.642).

**Figure 6:**
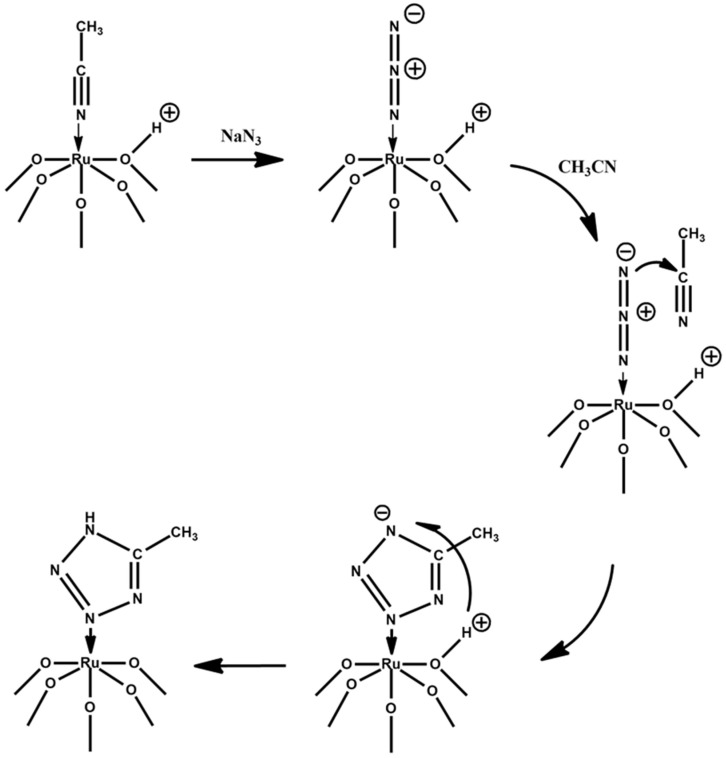
The proposed mechanism of N_3_^−^ and CH_3_CN coupling in the coordination sphere of Ru.

We can conclude that activation of azide ligand in the coordination sphere of Ru leads to a fast reaction with CH_3_CN into the tetrazole molecule. We proposed the following mechanism of this reaction (Figure 6).

In the CH_3_CN solution, and in the presence of excess azide, there are competing reactions leading to **Ru-N_3_** and **Ru-CH_3_CN** complexes. These are not possible in CH_3_NO_2_ when stoichiometric amounts of azide are employed. 

The azide–alkyne Huisgen cycloaddition is a 1,3-dipolar cycloaddition between an azide and a terminal or internal alkyne to give a 1,2,3-triazole [49]. Typically, such reactions can be catalyzed by Cu(I) [50], Ru [51,52], and Ag(I) [53,54]. The Ru-based catalysts are typically low-valent organometallic complexes like [Cp*RuCl(PPh_3_)_2_], [Cp*RuCl(COD)], and [Cp*RuCl(NBD)] [52]. The ruthenium-catalyzed azide−alkyne cycloaddition proceeds by an oxidative coupling of the azide and alkyne to give a six-membered ruthenacycle intermediate, in which the first new carbon−nitrogen bond is formed between the more electronegative carbon of the alkyne and the terminal, electrophilic nitrogen of the azide. In our case POM plays the role of a bulky ligand with “hard” oxo-environment, and ruthenium has 3+ oxidation state. This nonfavorable arrangement can induce partial leaving of a noble metal atom from the POM lacuna to achieve the suitable geometry for oxidative addition. The above-mentioned mechanism does not include changing of the Ru coordination environment and can be more favorable.

Moreover, analysis of the HR-ESI-MS data reveals another product in the reaction mixture in CH_3_NO_2_. The *m*/*z* calculations suggest coordination of the {PW_11_O_39_Ru} moiety with a ligand of molecular weight between 28 and 30, which can be addressed to CO, NO, or N_2_. However, CH_3_NO_2_ cannot be a source of NO or CO under reaction conditions and most likely is not involved in the reaction. We are to assume, therefore, that this ligand comes from azide, and can be nothing except N_2_, attached to Ru^II^ (Figure 5). To confirm this, we studied the bulk solid mixture with Raman spectroscopy and found a band at 2135 cm^−1^ (Figure S22). Stable Ru^II^ complexes with coordinated N_2_ molecule have been reported since the 1960s, including the first examples of dinitrogen complexes, isolated as [Ru(NH_3_)_5_N_2_]X_2_ (X = Br^−^, I^−^, BF_4_^−^, PF_6_^−^) [55]. According to the Raman data, the N_2_ stretches can lie between 2103 cm^−1^ (for *trans*-[RuN_3_(N_2_)(en)_2_]PF_6_ [56]) and 2140 cm^−1^ (for [Ru_2_H_6_N_2_(PPh_3_)_4_] [57]). Therefore, based on the HR-ESI-MS and Raman data, we can suggest formation of [PW_11_O_39_{Ru^II^(N_2_)}]^5−^ complex following N_3_^−^ activation upon coordination to the {PW_11_O_39_Ru}. The formation of the [PW_11_O_39_{Ru^II^(N_2_)}]^5−^ can be rationalized by the following simplified scheme:

{**Ru-CH_3_CN**} + N_3_^–^ = {**Ru-N_3_**} + CH_3_CN (1)

{**Ru-N_3_**} = {**Ru-N**} + N_2_ (2)

{**Ru-N**} + {**Ru-N_3_**} = 2{**Ru-N_2_**} (3)

After detection of **Ru-N_2_** complex in the mixture of products isolated after the reaction in CH_3_NO_2_, we checked a mixture isolated from acetonitrile and found peaks from the same complex with nitrogen. This means that heating of **Ru-CH_3_CN** with azide induces two parallel reactions of activated azide, resulting in tetrazole formation or nitrogen stabilization. The [PW_11_O_39_Ru^III^(N_3_)]^5−^ complex must be considered as a highly reactive intermediate.

## 3. Materials and Methods

K_2_[RuNOCl_5_] was prepared according to the literature [58]. IR spectra were recorded on a FT-801 FT-IR spectrometer (Simex, Russia). Elemental analysis was carried out on a Eurovector EA 3000 CHN analyzer. The TGA measurements were performed on a NETZSCH TG 209 F3 thermobalance in aluminum crucibles while heating the samples from 30 to 300 °C at a step of 10 °C. A 1.5 kW full-spectrum Hg lamp (GO Pnik, Iskitim, Russia) was used for the bulk solution photolysis. 

The cyclic voltammograms (CV) were recorded with a 797 VA Computrace system (Metrohm, Zurich, Switzerland). All measurements were performed with a conventional three-electrode configuration consisting of glassy carbon working and platinum auxiliary electrodes and an Ag/AgCl/KCl reference electrode. The solvent used in all experiments was CH_3_CN, which was deoxygenated before use. Tetra-n-butylammonium hexafluorophosphate (0.1 M solution) was used as a supporting electrolyte. The concentration of the complexes was approximately 10^−3^ M. The potential scan rate was 100 mV/s. The half-wave potential (E_1/2_) values were determined as (E_a_ + E_c_)/2, where E_a_ and E_c_ refer to anodic and cathodic peak potentials, respectively. Ferrocene was used as an internal standard, and the Fc/Fc^+^ potential was 0.43 V.

The high-resolution electrospray ionization mass spectrometric (HR-ESI-MS) measurements were performed at the Center of Collective Use “Mass Spectrometric Investigations” SB RAS (Novosibirsk, Russia). Spectra were obtained with a direct injection of liquid samples on an ESI quadrupole time-of-flight (ESI-q-TOF) high-resolution mass spectrometer Maxis 4G (Bruker Daltonics, Bremen, Germany). The spectra were recorded in the 300–3000 *m*/*z* range in negative mode.

Raman spectra were recorded on a LabRAM Horiba spectrometer (Horiba, Kyoto, Japan). An ion He-Ne laser (Simex, Moscow, Russia) with a wavelength of exciting light of 633 nm was used. The spectra were obtained in the backscattering geometry using a Raman microscope.

**Synthesis of (Bu_4_N)_4_[PW_11_O_39_{Ru(NO)}] (Ru-NO)**: 1 g (0.31 mmol) K_7_[PW_11_O_39_]·14H_2_O was dissolved in 15 mL H_2_O. Then, the solution of 0.132 g (0.31 mmol) K_2_[RuNOCl_5_] in 1 mL H_2_O was added. The resulting mixture was transferred into a Teflon-lined Parr autoclave and kept at 150 °C for 18 h. After cooling to the room temperature, TBABr was added (1 g, 0.95 mmol). The precipitation was filtered on a glass filter and rinsed with 200 mL of a distilled water. Yield: 1.070 g (95%). ^31^P NMR (CH_3_CN + CD_3_CN): −13.88 ppm. (Figure S23). IR (ATR, cm^−1^): 1846(s), 1482 (m), 1462 (m), 1380 (w), 1151 (w), 1089 (m), 1036 (m), 956 (s), 884 (m), 793 (vs), 658 (m), 613 (w), 606 (w), 590 (w), 578 (w), 569 (w), 554 (w). **EA**, found C,H,N (%): 16.4, 3.1, 1.5; calc C,H,N (%): 16.3, 3.1, 1.6.

**Synthesis of (Bu_4_N)_4_[PW_11_O_39_{Ru(CH_3_CN)}] (Ru-CH_3_CN)**: 0.1 g (0.028 mmol) of (Bu_4_N)_4_[PW_11_O_39_{Ru(NO)}] was dissolved in 10 mL of CH_3_CN. (Bu_4_N)_4_[PW_11_O_39_{Ru(CH_3_CN)}] was generated by photolysis for 90 min. The solid product was obtained by precipitation with diethyl ether. Yield: 0.07 g (64%). ^31^P NMR (CH_3_CN + CD_3_CN): −13.57 ppm. (Figure S24). IR (ATR, cm^−1^): 1482 (m), 1465 (m), 1378 (w), 1152 (w), 1079 (m), 1045 (m), 954 (s), 879 (s), 784 (vs), 626 (m), 613 (m), 598 (w), 587 (m), 578 (w), 563 (w), 556 (w). **EA**, found C,H,N (%): 20.8, 3.6, 2.0; calc C,H,N (%): 20.9, 3.9, 1.8.

**Reaction of Ru-CH_3_CN with NaN_3_ in acetonitrile:** Crude sodium azide (0.017 g, 0.26 mmol) was added to the solution of freshly prepared (Bu_4_N)_4_[PW_11_O_39_{Ru(CH_3_CN)}] (0.100 g, 0.026 mmol) in 10 mL of CH_3_CN. The reaction mixture was kept at 70 °C upon stirring for 18 h. After cooling to room temperature, white precipitate was filtered off. The resulting product was isolated by vapor diffusion of Et_2_O. Yield: 0.70 g.

**Reaction of Ru-CH_3_CN with NaN_3_ in nitromethane**: 0.120 g (0.032 mmol) of (Bu_4_N)_4_[PW_11_O_39_{Ru(CH_3_CN)}] was dissolved in 7 mL of CH_3_NO_2_. Then, 0.044 g (0.64 mmol) of solid NaN_3_ was added to the solution. The resulting mixture was transferred into a Teflon-lined Parr autoclave and kept at 100 °C for 18 h. The formed precipitate was filtered off, and the reaction product was isolated by the addition of diethyl ether. Yield: 0.092 g. IR (ATR, cm^−1^): 1477 (m), 1469 (m), 1376 (w), 1150 (w), 1077 (s), 1047 (m), 970 (s), 955 (s), 888 (sh), 880 (s), 798 (sh), 784 (vs), 773 (vs), 657 (s), 627 (s), 594 (m), 580 (m), 559 (m). 

## 4. Conclusions

This manuscript summarized our studies of Ru-atom reactivity inside the POM backbone toward azide anion. We detected two reaction pathways resulting in (i) azide–acetonitrile click reaction and (ii) azide decomposition. The first one produces coordinated tetrazole and the second generates a complex with coordinated N_2_. Such reactivity is important for organic substrate transformation and N_2_ activation. Further studies in these directions are in progress.

## Supplementary Materials

The following are available online at https://www.mdpi.com/1420-3049/25/8/1859/s1, Figure S1: Full spectrum of **Ru-NO** (calculated patterns have negative intensities), Figure S2: Zoomed 1637–1661 *m*/*z* region of spectrum of **Ru-NO** (calculated isotopic patterns have negative intensities), Figure S3: Zoomed 931–942 *m*/*z* and 1011–1023 *m*/*z* regions of spectrum of **Ru-NO** (calculated isotopic patterns have negative intensities), Table S1: Peak assignment for ESI-MS spectrum of **Ru-NO**, Figure S4: Full spectrum of **Ru-CH_3_CN** (calculated patterns have negative intensities), Table S2: Peak assignment for ESI-MS spectrum of **Ru-CH_3_CN**, Figure S5: Zoomed 1643–1670 *m*/*z* region of spectrum of **Ru-CH_3_CN** (calculated isotopic patterns have negative intensities), Figure S6: Zoomed 1014–1027 *m*/*z* region of spectrum of **Ru-CH_3_CN** (calculated isotopic patterns have negative intensities), Figure S7: The FT-IR of **Ru-NO** (gray), **Ru-CH_3_CN** (red) and **Ru-Tz** + **Ru-N_3_** (blue), Figure S8: Zoomed 1200–550 cm^–1^ region of IR spectra, Figure S9: Spectrum of **Ru-Tz** + **Ru-N_3_** (black) and 1st derivative (red), Figure S10: Full spectrum of **Ru-N_3_** and **Ru-CH_3_CN** (calculated patterns have negative intensities), Figure S11: Zoomed 949–968 *m*/*z* region of spectrum of **Ru-N_3_** and **Ru-CH_3_CN** (calculated isotopic patterns have negative intensities), Figure S12: Zoomed 1011–1053 *m*/*z* region of spectrum of **Ru-N_3_** and **Ru-CH_3_CN** (calculated isotopic patterns have negative intensities), Figure S13: Zoomed 1108–1123 *m*/*z* region of spectrum of **Ru-N_3_** and **Ru-CH_3_CN** (calculated isotopic patterns have negative intensities), Figure S14: Zoomed 1644–1695 *m*/*z* region of spectrum of **Ru-N_3_** and **Ru-CH_3_CN** (calculated isotopic patterns have negative intensities), Figure S15: Zoomed 1786–1804 *m*/*z* region of spectrum of **Ru-N_3_** and **Ru-CH_3_CN** (calculated isotopic patterns have negative intensities), Table S3: Peak assignment for ESI-MS spectrum of **Ru-N_3_** and **Ru-CH_3_CN**, Figure S16: Full spectrum of **Ru-N3** and **Ru-Tz** (calculated patterns have negative intensities), Figure S17: Zoomed 930–961 *m*/*z* region of spectrum of **Ru-N_3_** and **Ru-Tz** (calculated isotopic patterns have negative intensities), Figure S18: Zoomed 1011–1036 *m*/*z* region of spectrum of **Ru-N_3_** and **Ru-Tz** (calculated isotopic patterns have negative intensities), Figure S19: Zoomed 1536–1550 *m*/*z* region of spectrum of **Ru-N_3_** and **Ru-Tz** (calculated isotopic patterns have negative intensities), Figure S20: Zoomed 1636–1691 *m*/*z* region of spectrum of **Ru-N_3_** and **Ru-Tz** (calculated isotopic patterns have negative intensities), Figure S21: Zoomed 1759–1776 *m*/*z* region of spectrum of **Ru-N_3_** and **Ru-Tz** (calculated isotopic patterns have negative intensities), Table S4: Peak assignment for ESI-MS spectrum of **Ru-N_3_** and **Ru-Tz**, Figure S22:. The Raman spectrum of the solid mixture isolated after the Reaction of **Ru-CH_3_CN** with NaN_3_, Figure S23: ^31^P NMR spectrum of (Bu4N)3H[PW11O39{Ru(NO)}] in the mixture of CH_3_CN + CD_3_CN (−13.88 ppm), Figure S24: ^31^P NMR spectrum of (Bu4N)4[PW11O39{Ru(CH3CN)}] in the mixture of CH_3_CN + CD_3_CN (−13.57 ppm).
