# Supplementary material for: Easy Ligand Activation in the Coordination Sphere of Ru inside the [PW11O39]7– Backbone
Source: Molecules. 2020 Apr 17;25(8):1859. doi: 10.3390/molecules25081859 (PMC7221517; doi:10.3390/molecules25081859)
Supplement: Supplementary file 1 [file molecules-25-01859-s001.pdf]

Article

Azide activation in the coordination sphere of Ru inside the $[PW_{11}O_{39}]^{7-}$ backbone

Anna A. Mukhacheva [1], Artem L. Gushchin [1], Vadim V. Yanshole [2], Pavel A. Abramov [1,3], and Maksim N. Sokolov [1*]

1 Nikolaev Institute of Inorganic Chemistry SB RAS, 3 Akad. Lavrentiev Ave., 630090, Novosibirsk, Russia;
2 International Tomography Center, Institutskaya str. 3a, 630090, Novosibirsk, Russia;
3 South Ural State University, Chelyabinsk, 454080, Russia
* Correspondence: caesar@niic.nsc.ru

Received: date; Accepted: date; Published: date

Supporting information

Table of content

Fig. S1. Full spectrum of **Ru-NO** (calculated patterns have negative intensities)....................................3

Fig. S2. Zoomed 1637-1661 m/z region of spectrum of **Ru-NO** (calculated isotopic patterns have negative intensities)..3

Fig. S3. Zoomed 931-942 m/z and 1011-1023 m/z regions of spectrum of **Ru-NO** (calculated isotopic patterns have negative intensities)..4

Table. S1. Peak assignment for ESI-MS spectrum of **Ru-NO**..4

Fig. S4. Full spectrum of **Ru-CH$_3$CN** (calculated patterns have negative intensities). 5

Table. S2. Peak assignment for ESI-MS spectrum of **Ru-CH$_3$CN** 5

Fig. S5. Zoomed 1643-1670 m/z region of spectrum of **Ru-CH$_3$CN** (calculated isotopic patterns have negative intensities)..6

Fig. S6. Zoomed 1014-1027 m/z region of spectrum of **Ru-CH$_3$CN** (calculated isotopic patterns have negative intensities)..6

Fig. S7. The FT-IR of **Ru-NO** (gray), **Ru-CH$_3$CN** (red) and **Ru-Hc** + **Ru-N$_3$** (blue). 7

Fig. S8. Zoomed 1200-550 cm–1 region of IR spectra. 7

Fig. S9. Spectrum of **Ru-Hc** + **Ru-N$_3$** (black) and 1st derivative (red). 8

Fig. S10. Full spectrum of **Ru-N$_3$** and **Ru-CH$_3$CN** (calculated patterns have negative intensities). 9

Fig. S11. Zoomed 949-968 m/z region of spectrum of **Ru-N$_3$** and **Ru-CH$_3$CN** (calculated isotopic patterns have negative intensities)..9

Fig. S12. Zoomed 1011-1053m/z region of spectrum of **Ru-N$_3$** and **Ru-CH$_3$CN** (calculated isotopic patterns have negative intensities)..10

Fig. S13. Zoomed 1108-1123 m/z region of spectrum of **Ru-N$_3$** and **Ru-CH$_3$CN** (calculated isotopic patterns have negative intensities)..10

Fig. S14. Zoomed 1644-1695 m/z region of spectrum of **Ru-N$_3$** and **Ru-CH$_3$CN** (calculated isotopic patterns have negative intensities)..11

Fig. S15. Zoomed 1786-1804 m/z region of spectrum of **Ru-N$_3$** and **Ru-CH$_3$CN** (calculated isotopic patterns have negative intensities)..11

Table. S3. Peak assignment for ESI-MS spectrum of **Ru-N_3** and **Ru-CH_3CN** 12

Fig.S16. Full spectrum of **Ru-N_3** and **Ru-Hc** (calculated patterns have negative intensities).................13

Fig. S17. Zoomed 930-961 m/z region of spectrum of **Ru-N_3** and **Ru-Hc** (calculated isotopic patterns have negative intensities). 13

Fig. S18. Zoomed 1011-1036 m/z region of spectrum of **Ru-N_3** and **Ru-Hc** (calculated isotopic patterns have negative intensities). 14

Fig. S19. Zoomed 1536-1550 m/z region of spectrum of **Ru-N_3** and **Ru-Hc** (calculated isotopic patterns have negative intensities). 14

Fig. S20. Zoomed 1636-1691 m/z region of spectrum of **Ru-N_3** and **Ru-Hc** (calculated isotopic patterns have negative intensities). 15

Fig. S21. Zoomed 1759-1776 m/z region of spectrum of **Ru-N_3** and **Ru-Hc** (calculated isotopic patterns have negative intensities). 15

Table. S4. Peak assignment for ESI-MS spectrum of **Ru-N_3** and **Ru-Hc**...16

Fig. S22. The Raman spectrum of the solid mixture isolated after the Reaction of **Ru-CH_3CN** with NaN_3. 16

Fig. S23. ^{31}P NMR spectrum of $(Bu_4N)_3H[PW_{11}O_{39}\{Ru(NO)\}]$ in the mixture of CH_3CN+CD_3CN (-13.88 ppm). 17

Fig. S24. ^{31}P NMR spectrum of $(Bu_4N)_4[PW_{11}O_{39}\{Ru(CH_3CN)\}]$ in the mixture of CH_3CN+CD_3CN (-13.57 ppm). 17

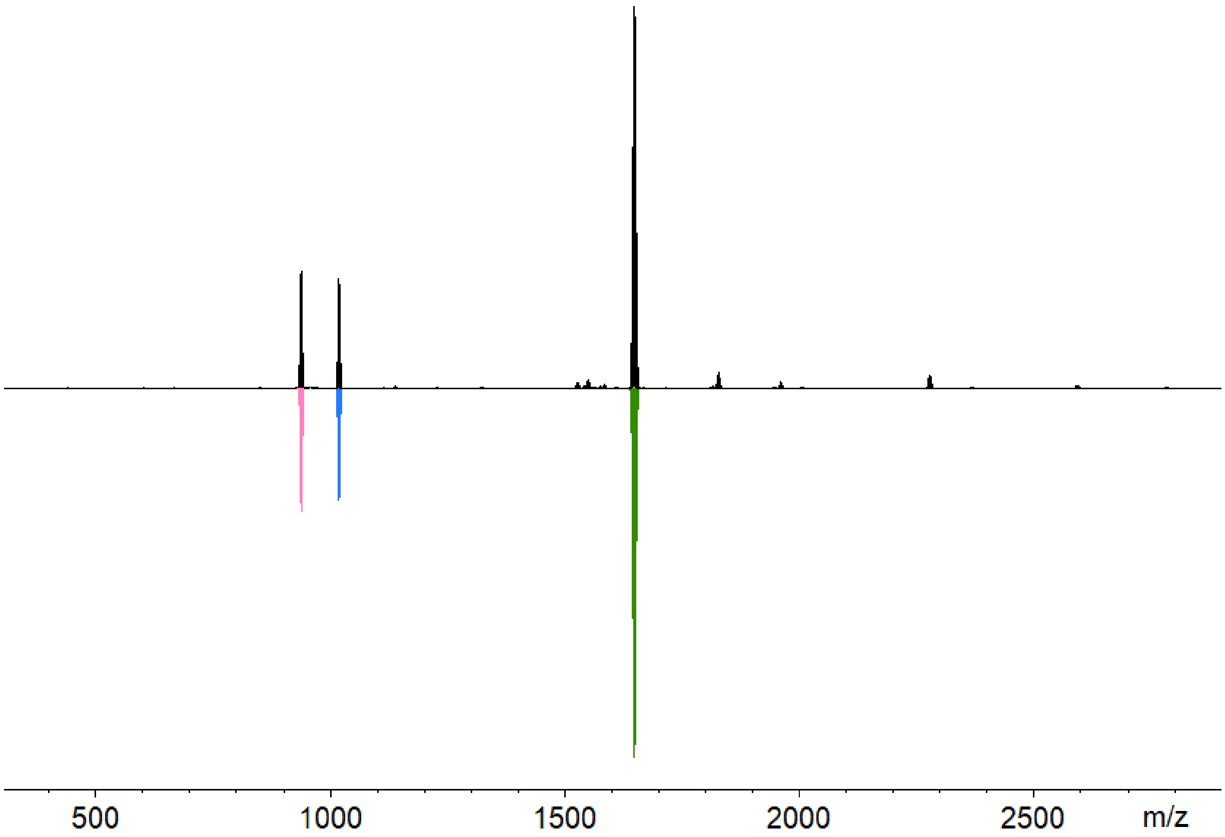

Fig. S1. Full spectrum of **Ru-NO** (calculated patterns have negative intensities).

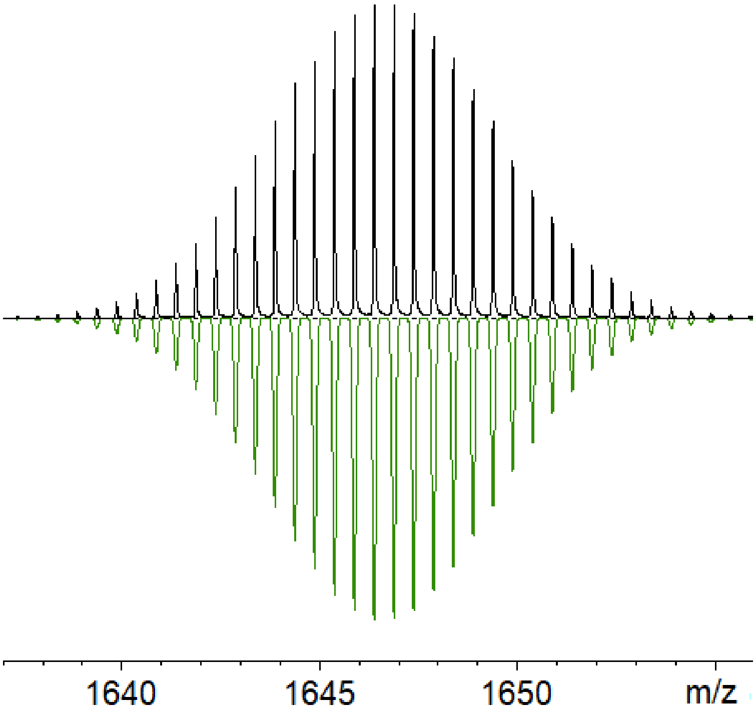

Fig. S2. Zoomed 1637-1661 m/z region of spectrum of **Ru-NO** (calculated isotopic patterns have negative intensities).

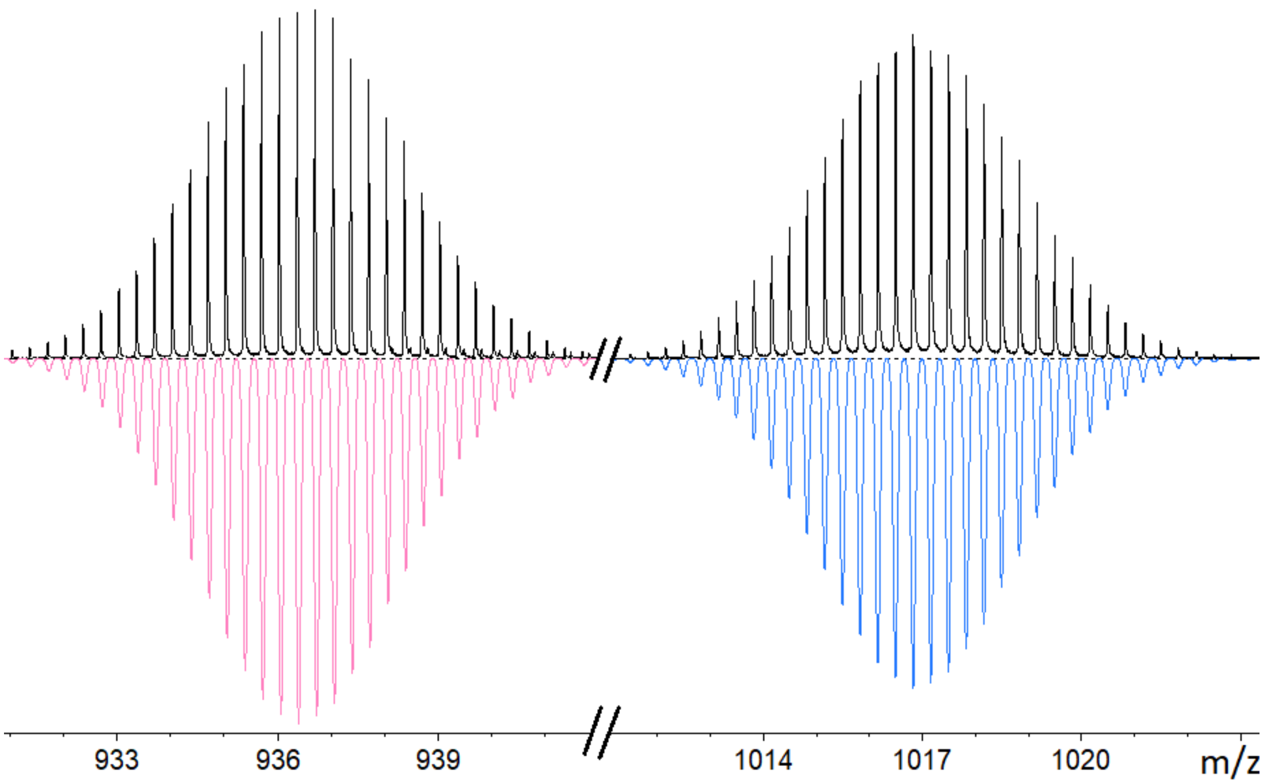

Fig. S3. Zoomed 931-942 m/z and 1011-1023 m/z regions of spectrum of **Ru-NO** (calculated isotopic patterns have negative intensities).

Table. S1. Peak assignment for ESI-MS spectrum of **Ru-NO**

**anion**	**exp (m/z)**	**calc (m/z)**
$H^+ + [PW_{11}O_{39}\{Ru(NO)\}]^{4-}$	936.4	936.4
$Bu_4N^+ + [PW_{11}O_{39}\{Ru(NO)\}]^{4-}$	1016.8	1016.8
$2Bu_4N^+ + [PW_{11}O_{39}\{Ru(NO)\}]^{4-}$	1646.6	1646.6

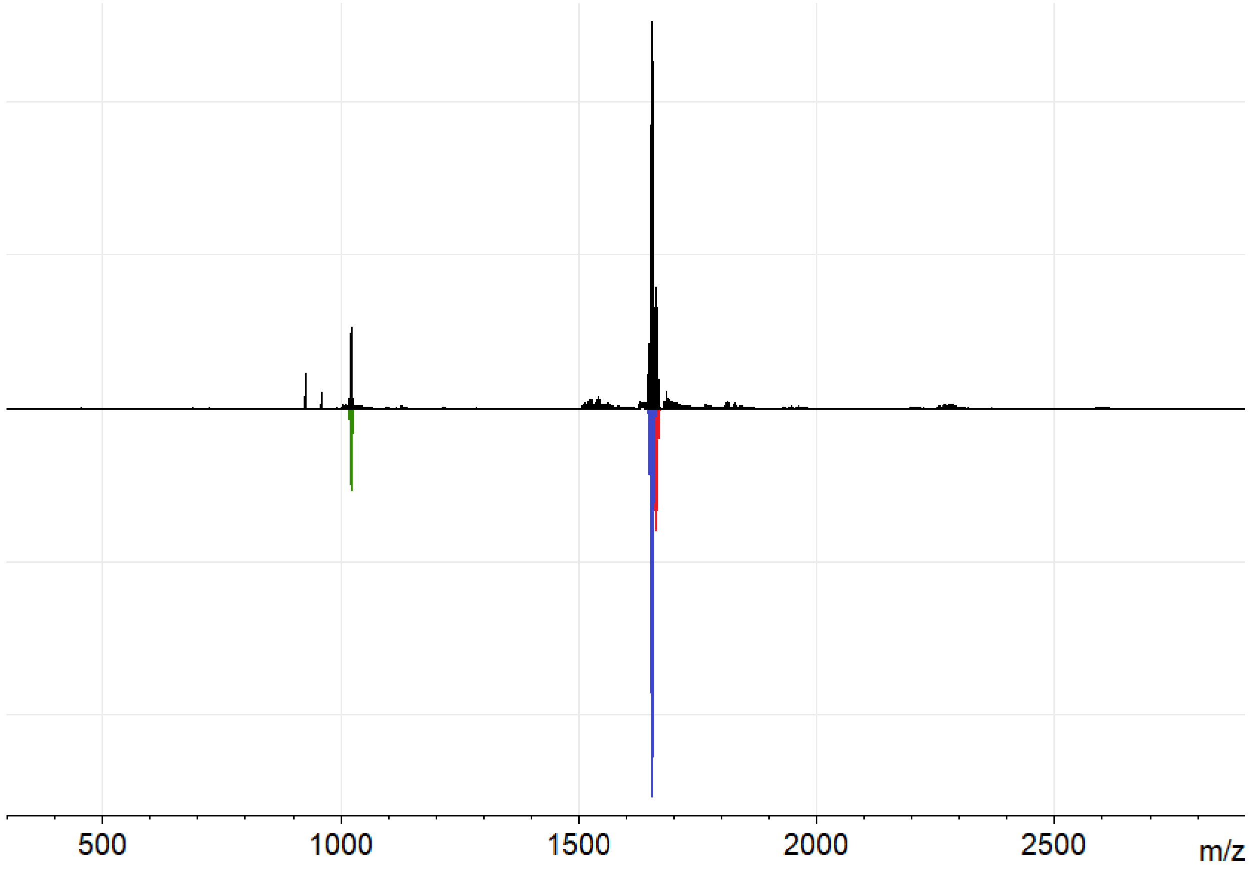

Fig. S4. Full spectrum of Ru-CH_3CN (calculated patterns have negative intensities).

Table. S2. Peak assignment for ESI-MS spectrum of Ru-CH_3CN

**anion**	**exp (m/z)**	**calc (m/z)**
$Bu_4N^+ + [PW_{11}O_{39}\{Ru(CH_3CN)\}]^{4-}$	1020.6	1020.6
$2Bu_4N^+ + [PW_{11}O_{39}\{Ru(CH_3CN)\}]^{4-}$	1652.1	1652.1
$2Bu_4N^+ + [PW_{11}O_{39}\{Ru(CH_3CN)\}]^{4-} + H_2O$	1661.1	1661.1

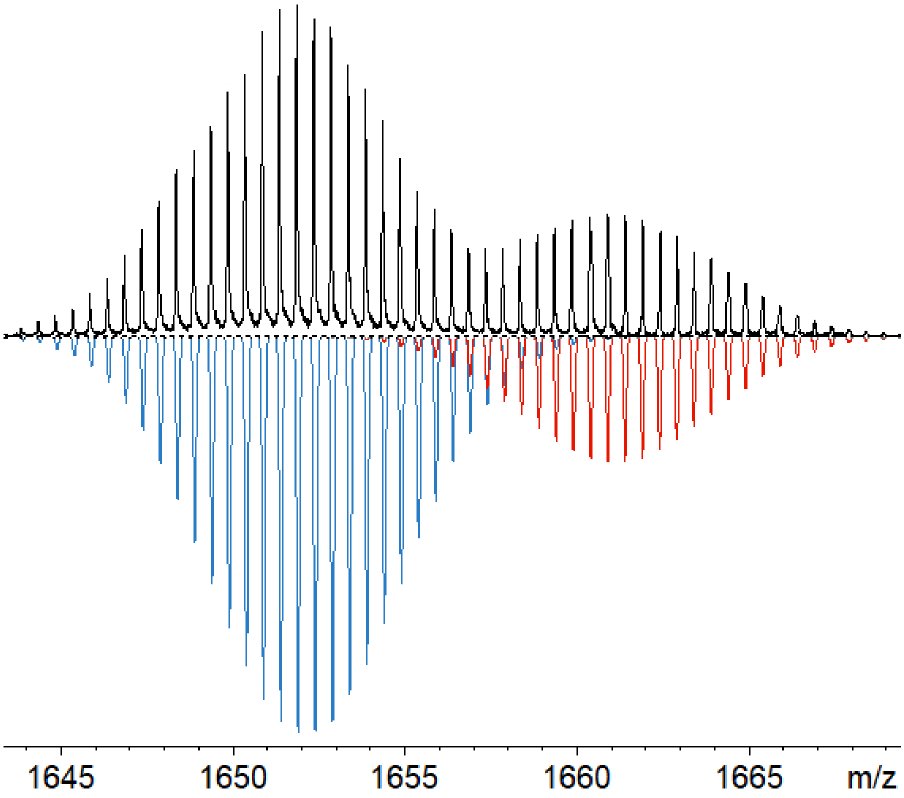

Fig. S5. Zoomed 1643-1670 m/z region of spectrum of Ru-CH_3CN (calculated isotopic patterns have negative intensities).

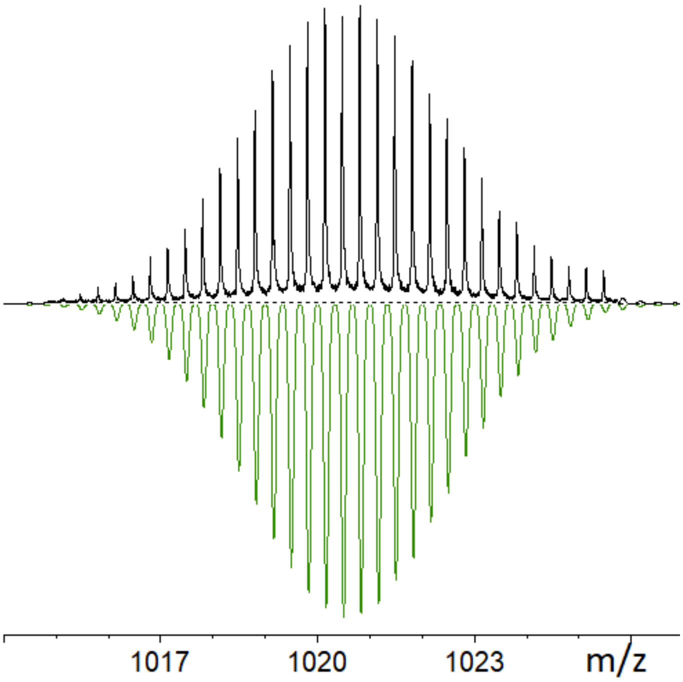

Fig. S6. Zoomed 1014-1027 m/z region of spectrum of Ru-CH_3CN (calculated isotopic patterns have negative intensities).

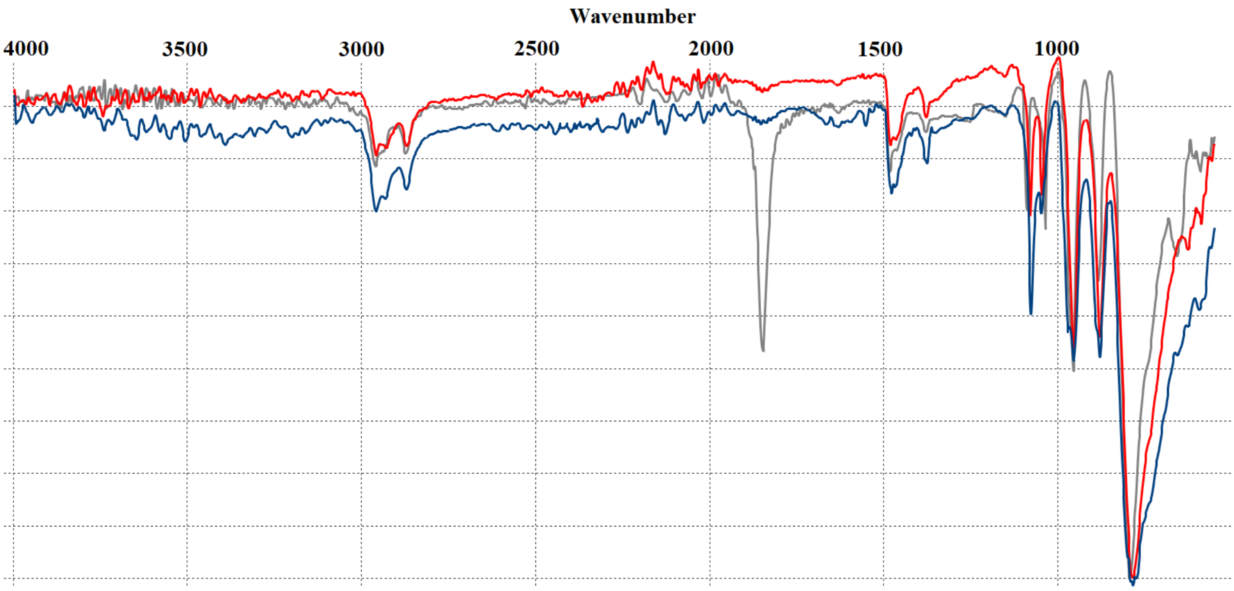

Fig. S7. The FT-IR of Ru-NO (gray), Ru-CH_3CN (red) and Ru-Hc + Ru-N_3 (blue).

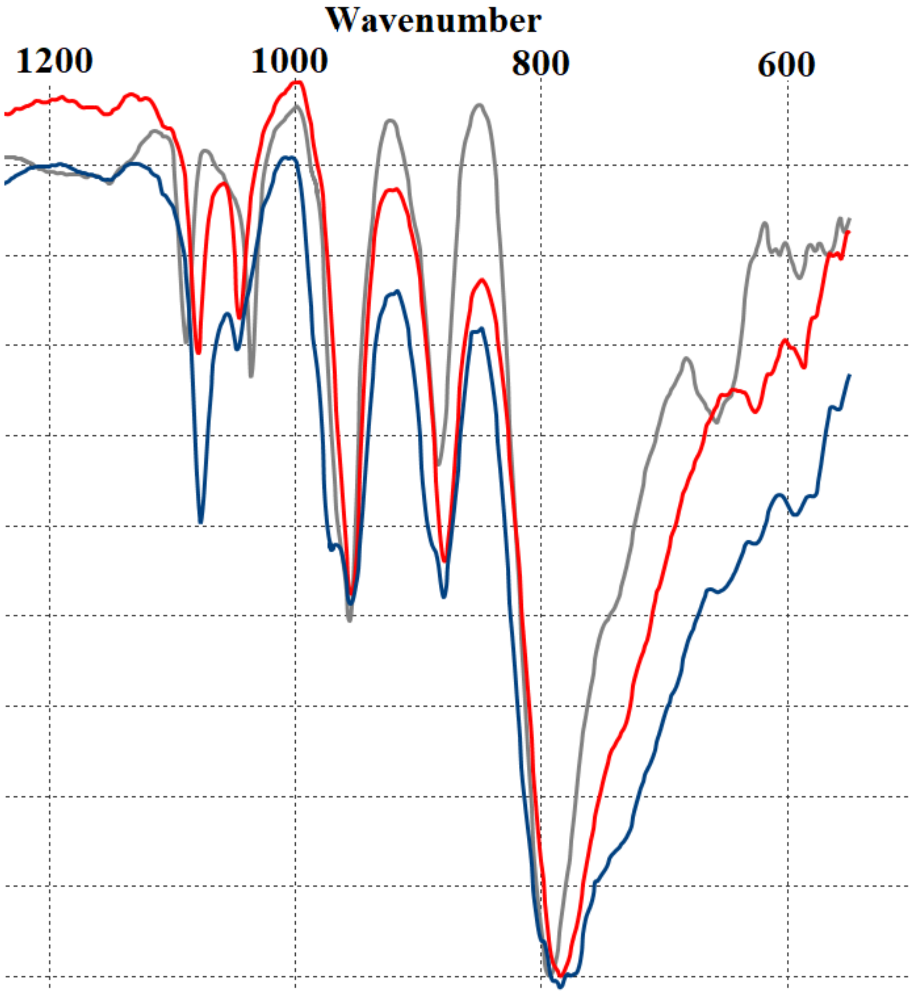

Fig. S8. Zoomed 1200-550 cm–1 region of IR spectra.

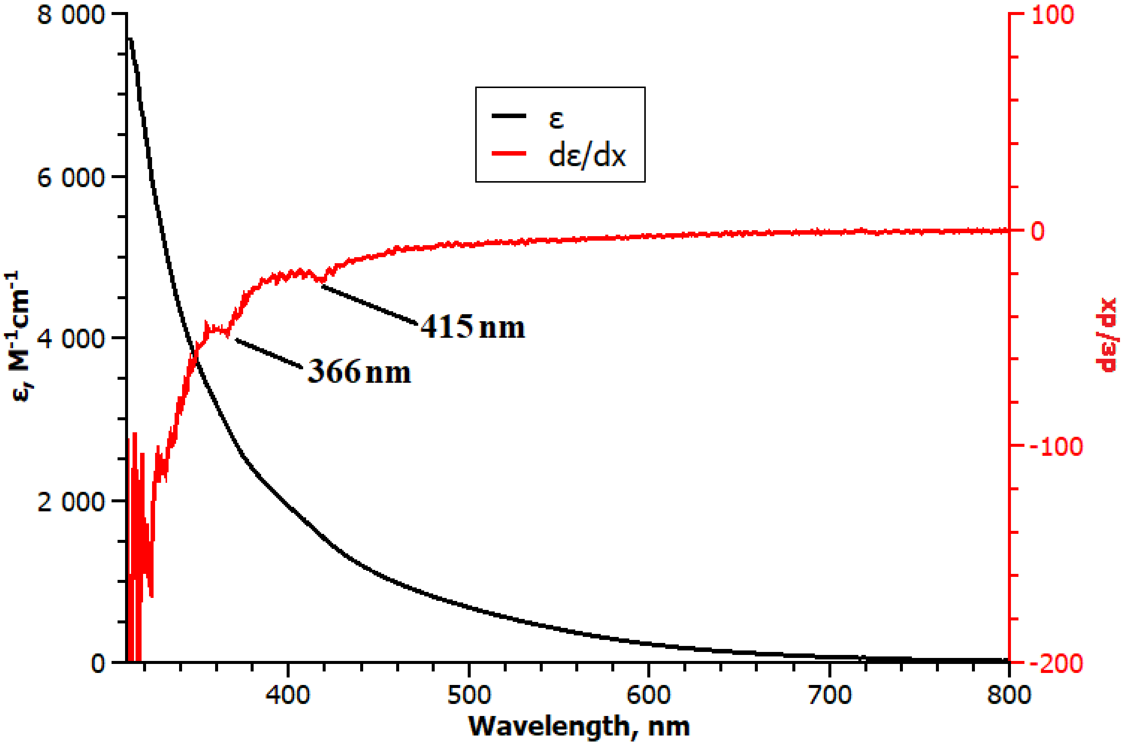

Fig. S9. Spectrum of Ru-Hc + Ru-N_3 (black) and 1st derivative (red).

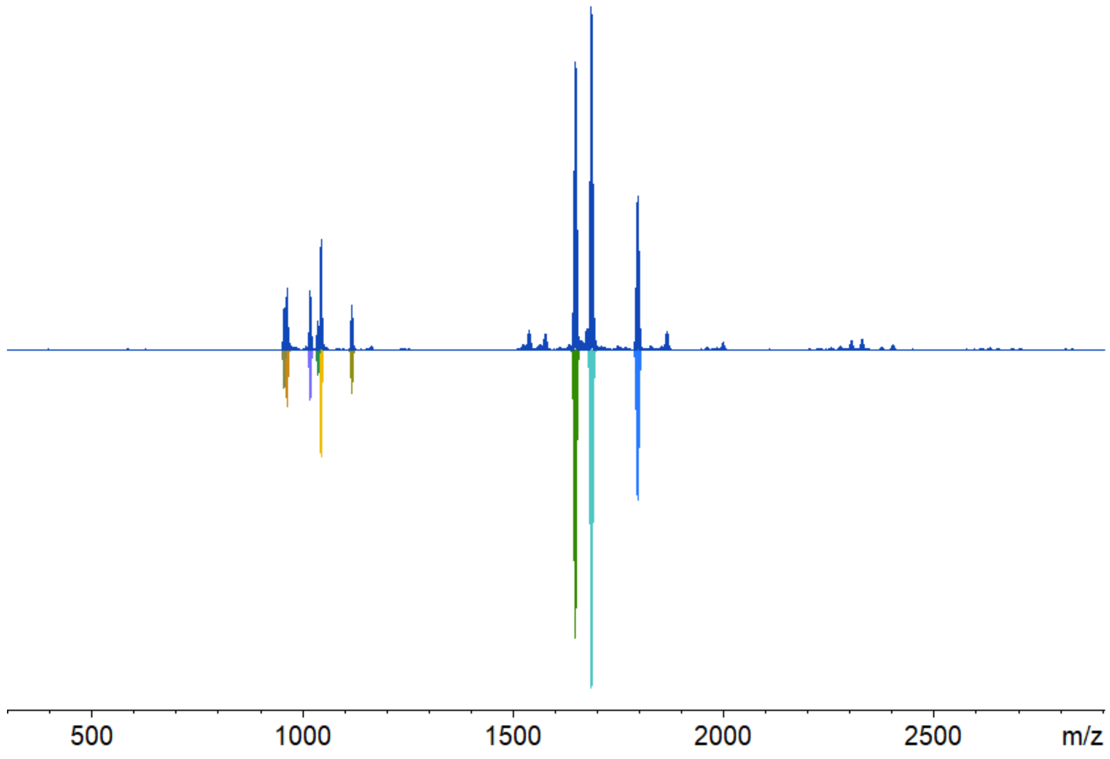

Fig. S10. Full spectrum of **Ru-N$_3$** and **Ru-CH$_3$CN** (calculated patterns have negative intensities).

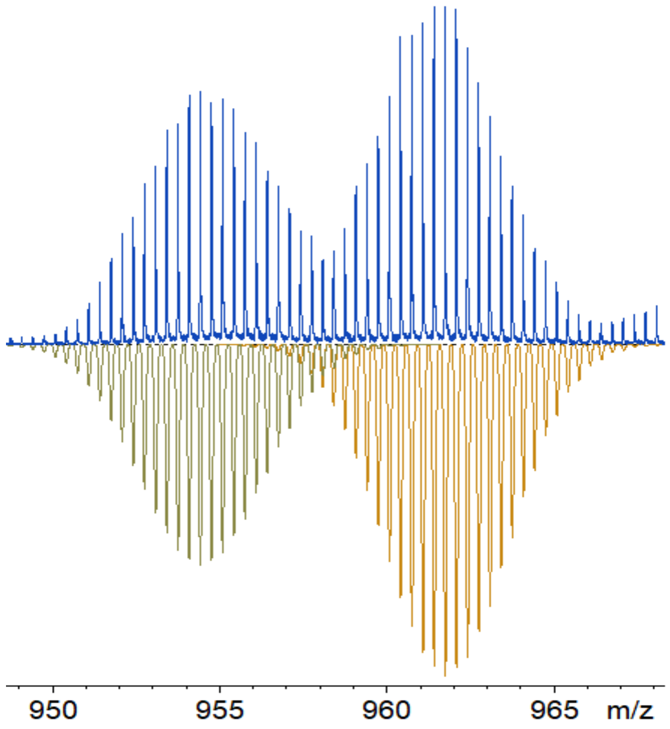

Fig. S11. Zoomed 949-968 m/z region of spectrum of **Ru-N$_3$** and **Ru-CH$_3$CN** (calculated isotopic patterns have negative intensities).

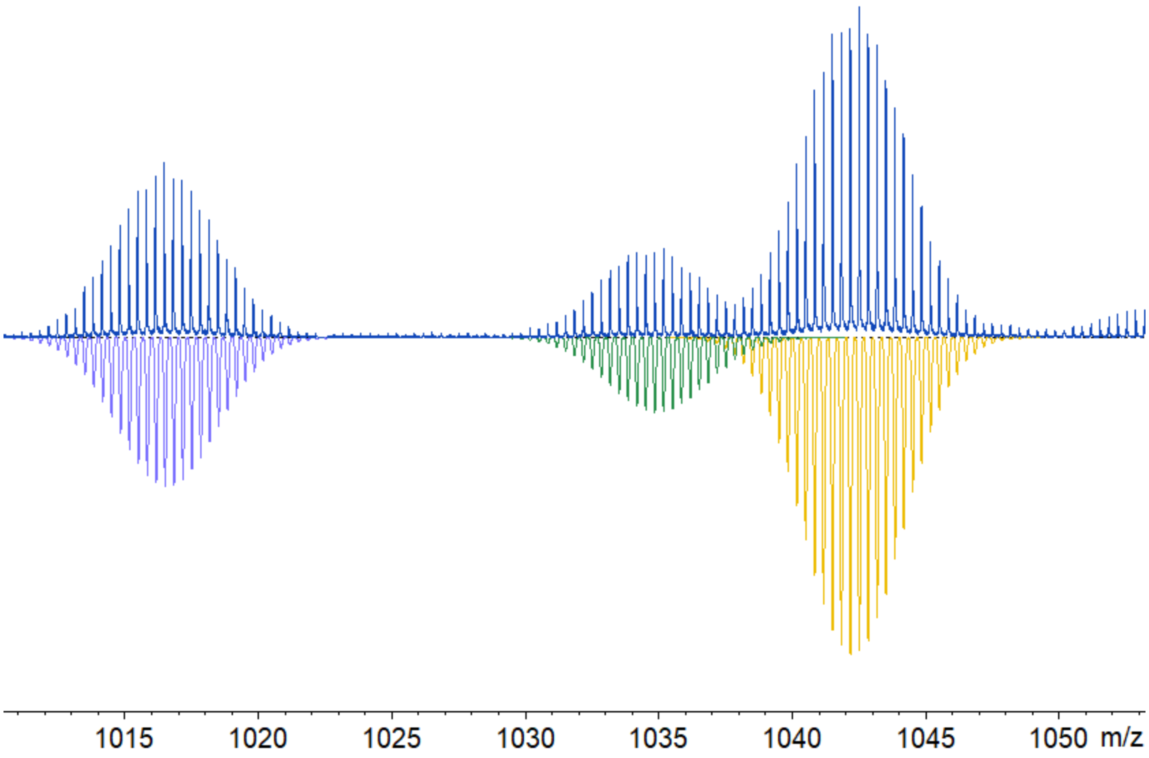

Fig. S12. Zoomed 1011-1053m/z region of spectrum of Ru-N_3 and Ru-CH_3CN (calculated isotopic patterns have negative intensities).

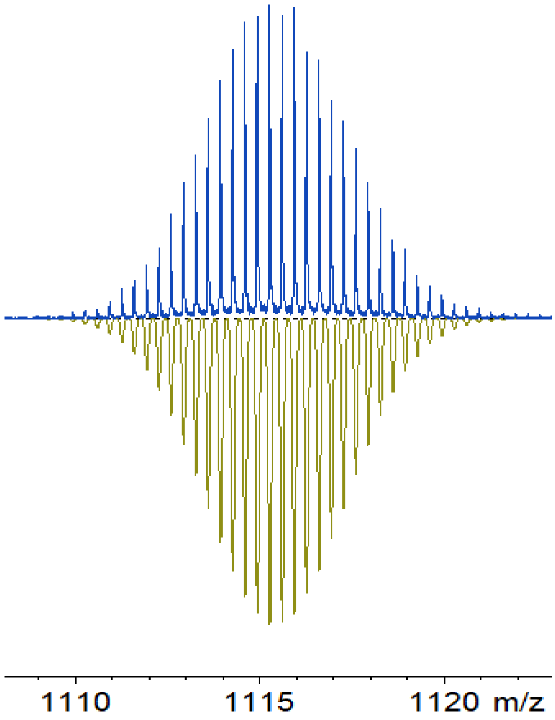

Fig. S13. Zoomed 1108-1123 m/z region of spectrum of Ru-N_3 and Ru-CH_3CN (calculated isotopic patterns have negative intensities).

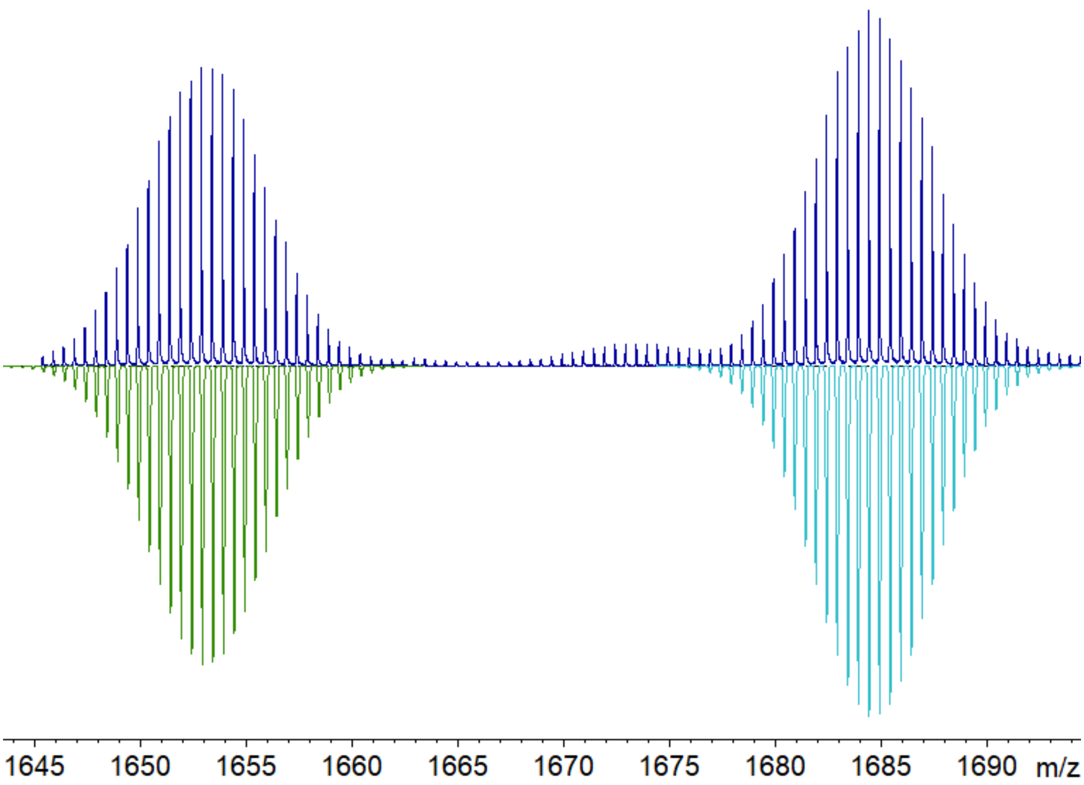

Fig. S14. Zoomed 1644-1695 m/z region of spectrum of Ru-N_3 and Ru-CH_3CN (calculated isotopic patterns have negative intensities).

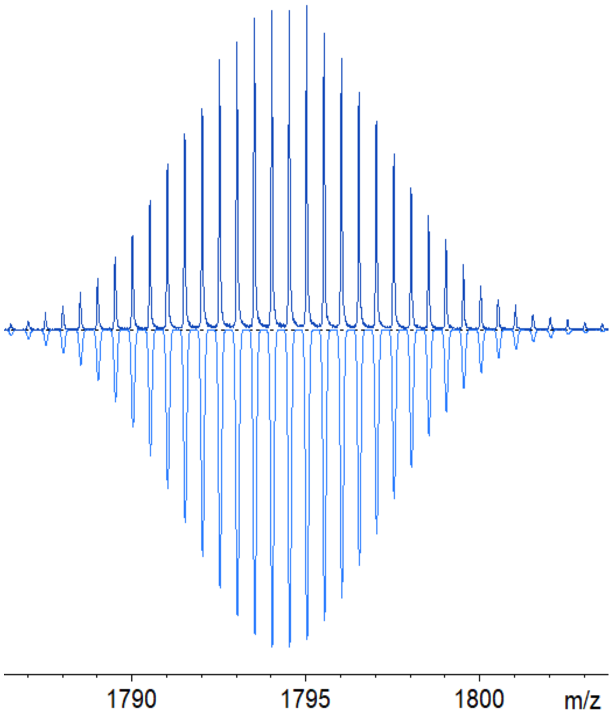

Fig. S15. Zoomed 1786-1804 m/z region of spectrum of Ru-N_3 and Ru-CH_3CN (calculated isotopic patterns have negative intensities).

Table. S3. Peak assignment for ESI-MS spectrum of **Ru-N$_3$** and **Ru-CH$_3$CN**

**anion**	**exp (m/z)**	**calc (m/z)**
$2H^{+}$ + $[PW_{11}O_{39}\{RuN_3\}]^{5-}$ + CH_3CN	954.5	954.5
Na^{+} + H^{+} + $[PW_{11}O_{39}\{RuN_3\}]^{5-}$ + CH_3CN	961.8	961.8
Bu_4N^{+} + $[PW_{11}O_{39}\{Ru(CH_3CN)\}]^{4-}$	1020.6	1020.6
Bu_4N^{+} + H^{+} + $[PW_{11}O_{39}\{RuN_3\}]^{5-}$ + CH_3CN	1034.9	1034.9
Bu_4N^{+} + Na^{+} + $[PW_{11}O_{39}\{RuN_3\}]^{5-}$ + CH_3CN	1042.3	1042.3
$2Bu_4N^{+}$ + $[PW_{11}O_{39}\{RuN_3\}]^{5-}$ + CH_3CN	1115.4	1115.4
$2Bu_4N^{+}$ + H^{+} + $[PW_{11}O_{39}\{RuN_3\}]^{5-}$	1653.1	1653.1
$2Bu_4N^{+}$ + Na^{+} + $[PW_{11}O_{39}\{RuN_3\}]^{5-}$ + CH_3CN	1684.6	1684.6
$3Bu_4N^{+}$ + $[PW_{11}O_{39}\{RuN_3\}]^{5-}$ + CH_3CN	1794.4	1794.4

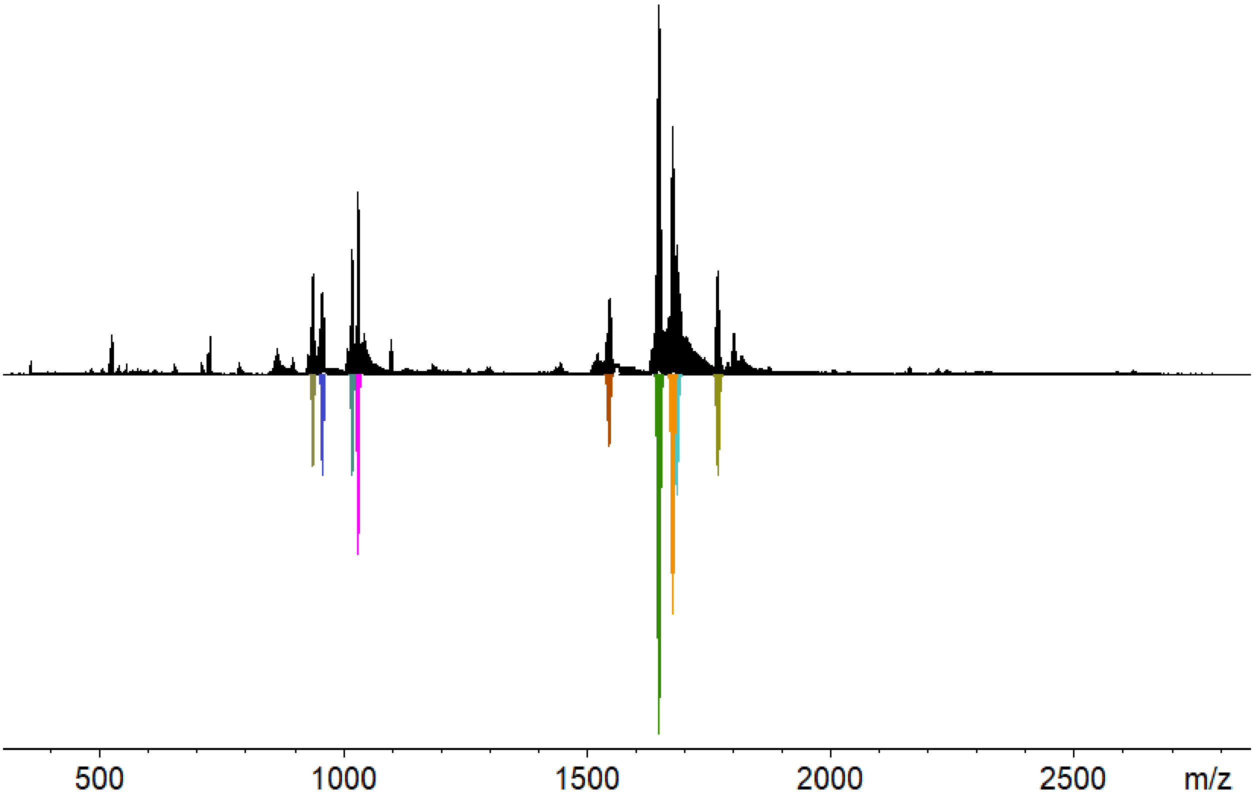

Fig.S16. Full spectrum of Ru-N_3 and Ru-Hc (calculated patterns have negative intensities).

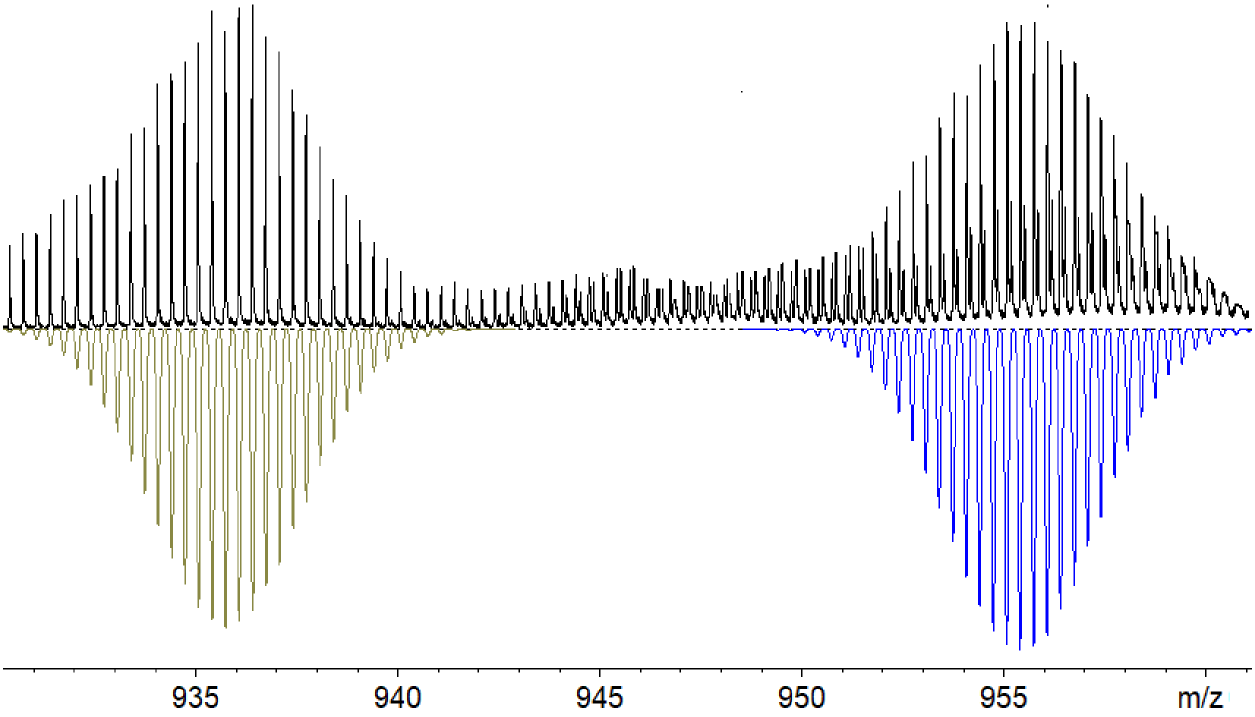

Fig. S17. Zoomed 930-961 m/z region of spectrum of Ru-N_3 and Ru-Hc (calculated isotopic patterns have negative intensities).

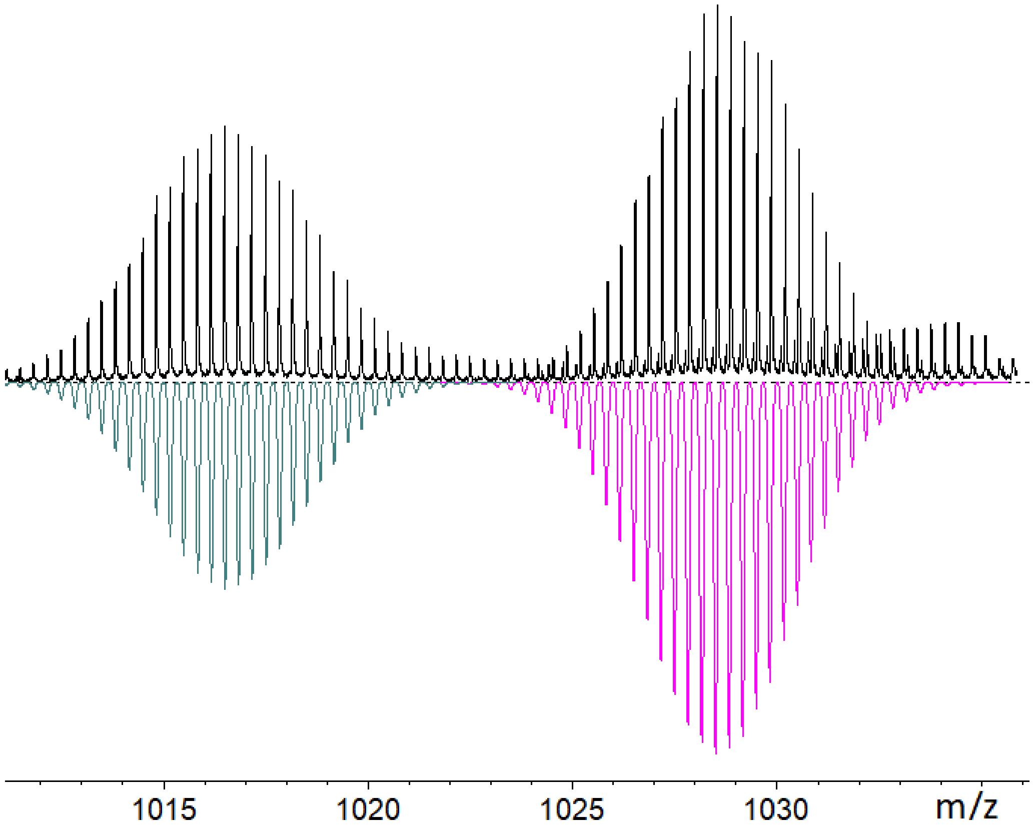

Fig. S18. Zoomed 1011-1036 m/z region of spectrum of Ru-N_3 and Ru-Hc (calculated isotopic patterns have negative intensities).

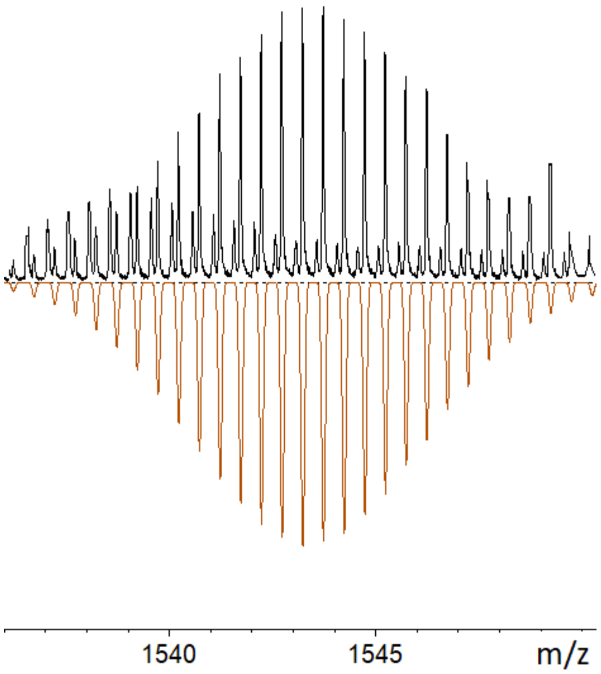

Fig. S19. Zoomed 1536-1550 m/z region of spectrum of Ru-N_3 and Ru-Hc (calculated isotopic patterns have negative intensities).

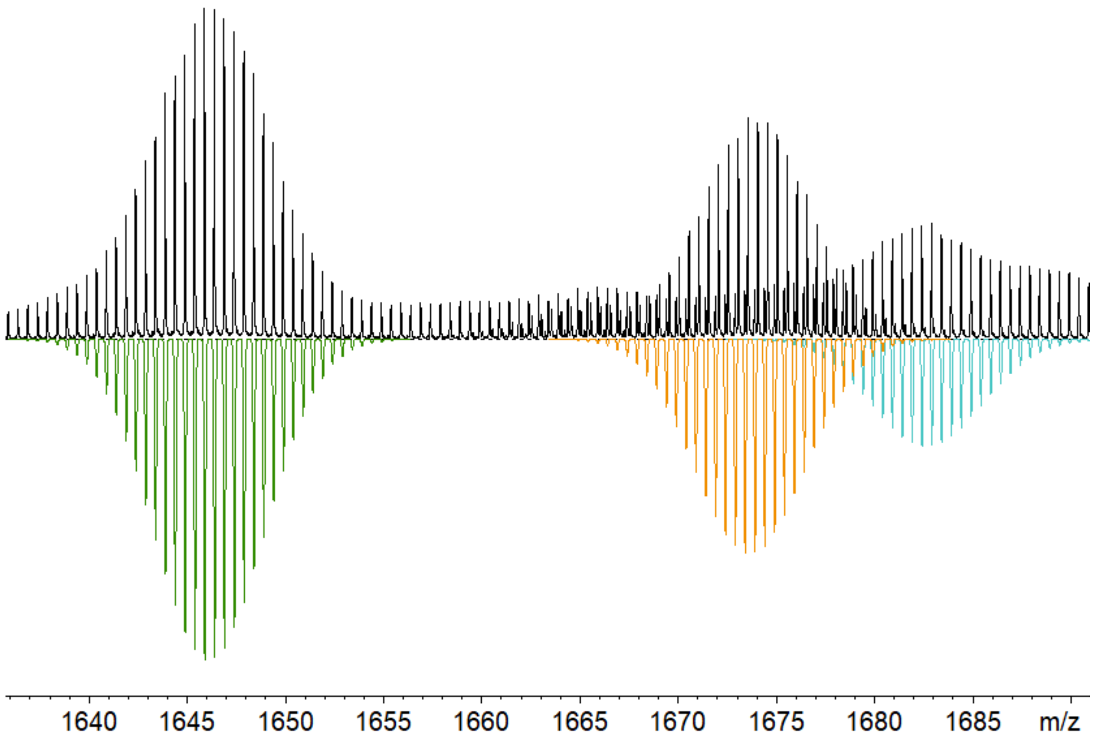

Fig. S20. Zoomed 1636-1691 m/z region of spectrum of Ru-N_3 and Ru-Hc (calculated isotopic patterns have negative intensities).

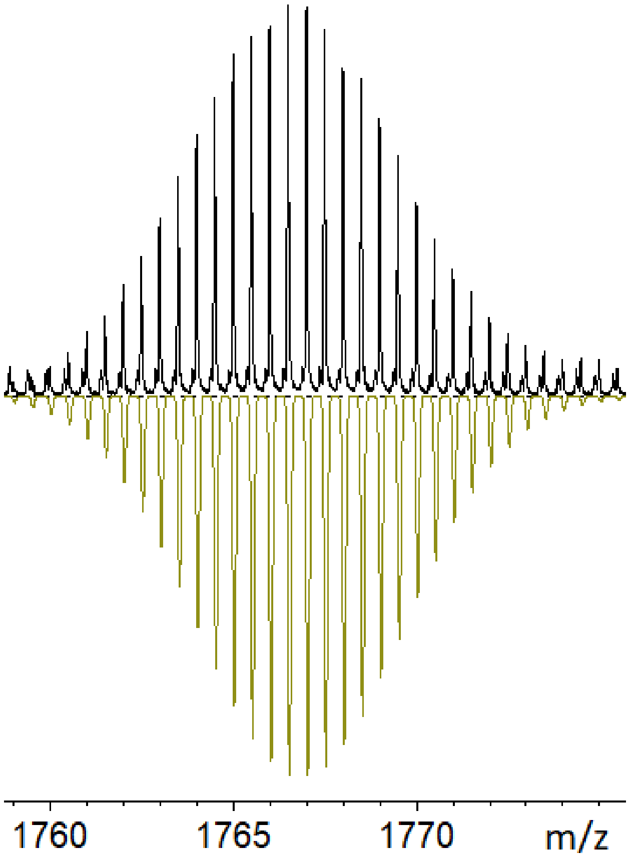

Fig. S21. Zoomed 1759-1776 m/z region of spectrum of Ru-N_3 and Ru-Hc (calculated isotopic patterns have negative intensities).

Table. S4. Peak assignment for ESI-MS spectrum of Ru-N_3 and Ru-Hc

anion	exp (m/z)	calc (m/z)
$2H^+ + [PW_{11}O_{39}\{RuN_2\}]^{5-}$	935.8	935.8
$2Na^+ + [PW_{11}O_{39}\{RuN_3\}]^{5-}$	955.4	955.4
$Bu_4N^+ + H^+ + [PW_{11}O_{39}\{RuN_2\}]^{5-}$	1016.6	1016.6
$Bu_4N^+ + Na^+ + [PW_{11}O_{39}\{RuN_3\}]^{5-}$	1028.6	1028.6
$Bu_4N^+ + Na^+ + H^+ + [PW_{11}O_{39}\{RuN_3\}]^{5-}$	1543.4	1543.4
$2Bu_4N^+ + H^+ + [PW_{11}O_{39}\{RuN_2\}]^{5-}$	1646.1	1646.1
$2Bu_4N^+ + [PW_{11}O_{39}\{Ru(Hc)\}]^{4-}$	1673.6	1673.6
$2Bu_4N^+ + [PW_{11}O_{39}\{Ru(Hc)\}]^{4-} + H_2O$	1682.6	1682.6
$3Bu_4N^+ + [PW_{11}O_{39}\{RuN_2\}]^{5-}$	1766.8	1766.8

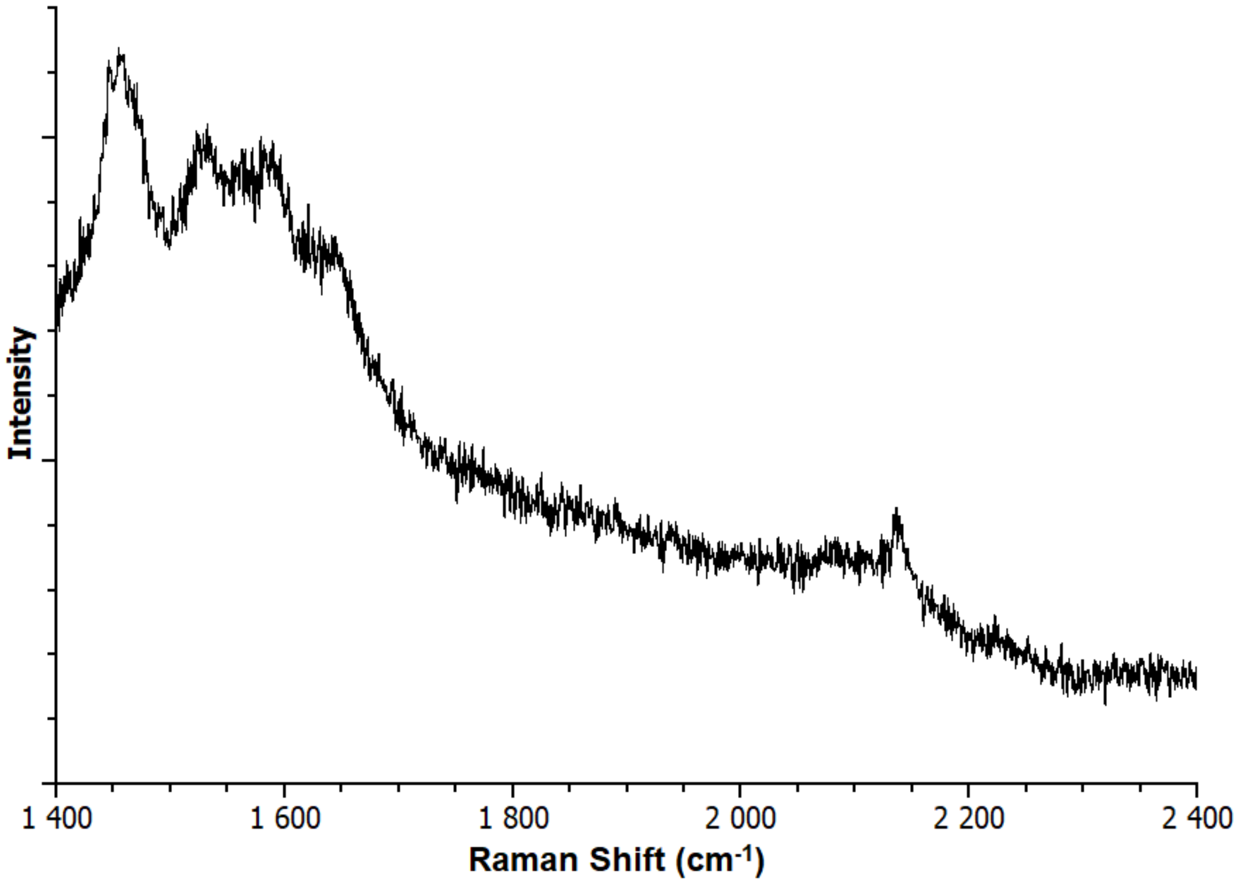

Fig. S22. The Raman spectrum of the solid mixture isolated after the Reaction of Ru-CH_3CN with NaN_3.

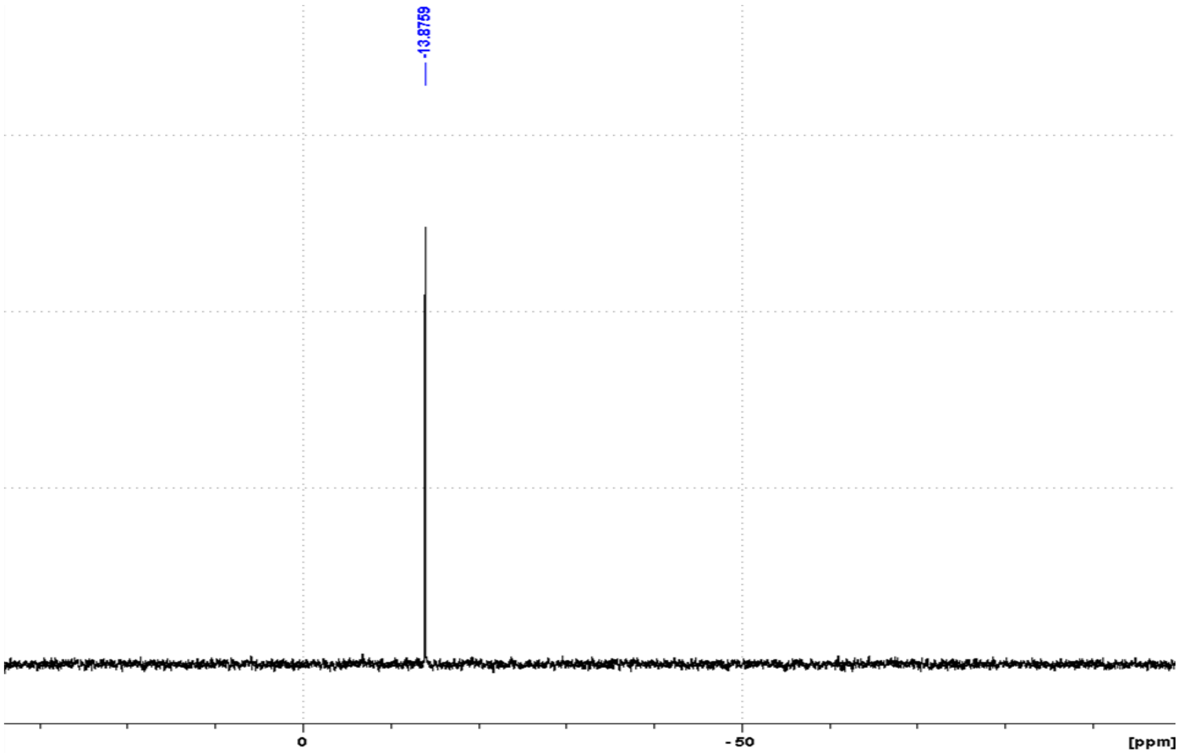

Fig. S23. ^{31}P NMR spectrum of $(Bu_4N)_3H[PW_{11}O_{39}\{Ru(NO)\}]$ in the mixture of CH_3CN+CD_3CN (-13.88 ppm).

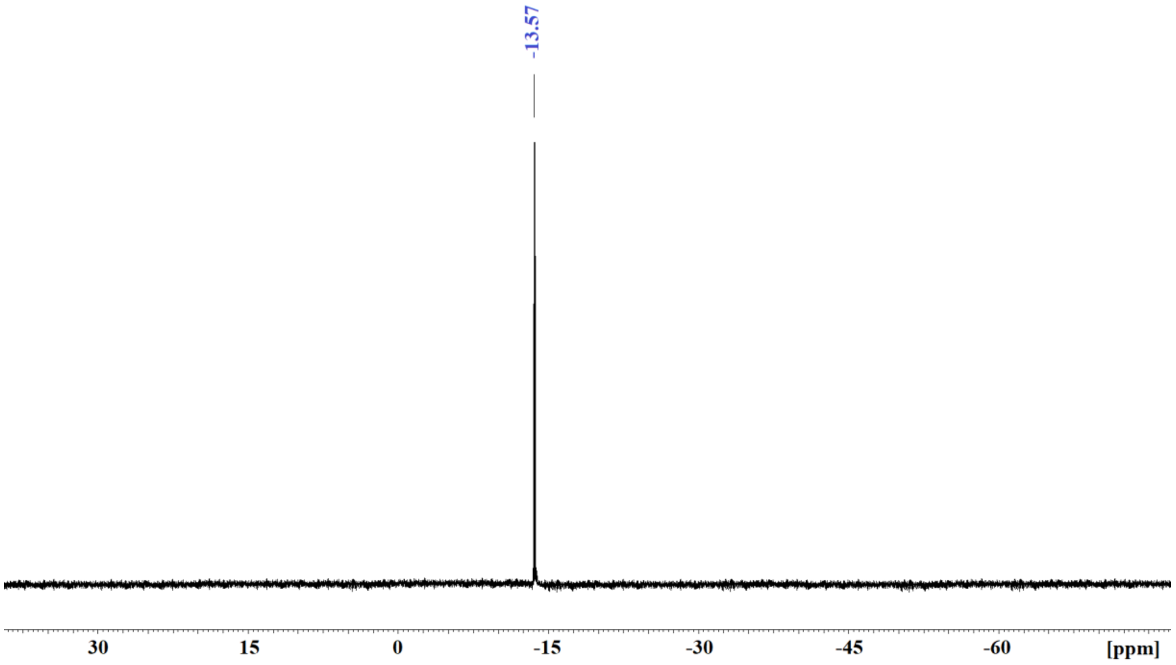

Fig. S24. ^{31}P NMR spectrum of $(Bu_4N)_4[PW_{11}O_{39}\{Ru(CH_3CN)\}]$ in the mixture of CH_3CN+CD_3CN (-13.57 ppm).
